# MDSCs use a complex molecular network to suppress T-cell immunity in a pulmonary model of fungal infection

**DOI:** 10.3389/fcimb.2024.1392744

**Published:** 2024-07-05

**Authors:** Valéria Lima Kaminski, Bruno Montanari Borges, Bianca Vieira Santos, Nycolas Willian Preite, Vera Lucia Garcia Calich, Flávio Vieira Loures

**Affiliations:** ^1^ Institute of Science and Technology, Federal University of São Paulo – UNIFESP, São Paulo, Brazil; ^2^ Department of Immunology, Institute of Biomedical Sciences, University of São Paulo – USP, São Paulo, Brazil

**Keywords:** MDSC, TLR2, TLR4, Dectin-1, paracoccidioidomycosis, PD-L1, IL-10, nitrotyrosine

## Abstract

**Background:**

Paracoccidioidomycosis (PCM) is a systemic endemic fungal disease prevalent in Latin America. Previous studies revealed that host immunity against PCM is tightly regulated by several suppressive mechanisms mediated by tolerogenic plasmacytoid dendritic cells, the enzyme 2,3 indoleamine dioxygenase (IDO-1), regulatory T-cells (Tregs), and through the recruitment and activation of myeloid-derived suppressor cells (MDSCs). We have recently shown that Dectin-1, TLR2, and TLR4 signaling influence the IDO-1-mediated suppression caused by MDSCs. However, the contribution of these receptors in the production of important immunosuppressive molecules used by MDSCs has not yet been explored in pulmonary PCM.

**Methods:**

We evaluated the expression of PD-L1, IL-10, as well as nitrotyrosine by MDSCs after anti-Dectin-1, anti-TLR2, and anti-TLR4 antibody treatment followed by *P. brasiliensis* yeasts challenge *in vitro*. We also investigated the influence of PD-L1, IL-10, and nitrotyrosine in the suppressive activity of lung-infiltrating MDSCs of C57BL/6-WT, Dectin-1KO, TLR2KO, and TLR4KO mice after *in vivo* fungal infection. The suppressive activity of MDSCs was evaluated in cocultures of isolated MDSCs with activated T-cells.

**Results:**

A reduced expression of IL-10 and nitrotyrosine was observed after *in vitro* anti-Dectin-1 treatment of MDSCs challenged with fungal cells. This finding was further confirmed *in vitro* and *in vivo* by using Dectin-1KO mice. Furthermore, MDSCs derived from Dectin-1KO mice showed a significantly reduced immunosuppressive activity on the proliferation of CD4^+^ and CD8^+^ T lymphocytes. Blocking of TLR2 and TLR4 by mAbs and using MDSCs from TLR2KO and TLR4KO mice also reduced the production of suppressive molecules induced by fungal challenge. *In vitro*, MDSCs from TLR4KO mice presented a reduced suppressive capacity over the proliferation of CD4^+^ T-cells.

**Conclusion:**

We showed that the pathogen recognition receptors (PRRs) Dectin-1, TLR2, and TLR4 contribute to the suppressive activity of MDSCs by inducing the expression of several immunosuppressive molecules such as PD-L1, IL-10, and nitrotyrosine. This is the first demonstration of a complex network of PRRs signaling in the induction of several suppressive molecules by MDSCs and its contribution to the immunosuppressive mechanisms that control immunity and severity of pulmonary PCM.

## 1 Introduction

Myeloid-derived suppressor cells (MDSCs) are heterogeneous cell populations that can impair immune responses. These cells comprise morphologically distinct subsets such as monocyte-like MDSCs (M-MDSCs) and polymorphonuclear-like MDSCs (PMN-MDSCs). M-MDSCs are classified as CD11b^+^Ly6G^−^Ly6C^hi^ and PMN-MDSCs are defined as CD11b^+^Ly6G^+^Ly6C^low^, both of which are implicated in various aspects of immune regulation, from pregnancy to diseases that involve chronic inflammation, including infections, autoimmune diseases, and other pathologies (Bronte et al., 2016; Köstlin et al., 2016; Goldmann et al., 2017). MDSCs have a strong ability to suppress T-cell responses. After migration to target sites that exhibit a milieu of cytokines, chemokines, and other inflammatory mediators, MDSCs adapt and acquire a suppressive behavior, such as the production of IDO-1, nitric oxide (NO), IL-10, as well as the expression of programmed death-ligand 1 (PD-L1), an immune checkpoint inhibitor (Gabrilovich and Nagaraj, 2009; Goh et al., 2013; Poe et al., 2013; Dorhoi and Du Plessis, 2017).

Characterized as a chronic systemic mycosis, Paracoccidioidomycosis (PCM) has a high prevalence in Latin America. The etiological agents of the disease are the thermally dimorphic fungi *Paracoccidioides brasiliensis*, *P. lutzii*, *P. americana*, *P. restrepiensis*, and *P. venezuelensis* (Teixeira et al., 2009; Turissini et al., 2017). The infection occurs from inhaling conidia or mycelial fragments into the lungs’ host, which can lead to a latent infection. However, the reactivation of a latent focus or the progression of a primary infection event can cause overt disease, which can be acute or chronic (McEwen et al., 1987; Brummer et al., 1993; Colombo et al., 2011; Coutinho et al., 2015; de Oliveira et al., 2016; Turissini et al., 2017). Both in human and murine models, resistance to PCM has been related to the predominant secretion of Th1 cytokines, such as interferon-gamma (IFN-γ), while the predominance of cytokines with a Th2 profile has been related to severe and progressive disease (Kashino et al., 2000; Souto et al., 2000; Pagliari et al., 2011; Pina et al., 2013). Furthermore, the expression of IL-17 in cells from patients with PCM has been associated with the organization of mucosal and cutaneous granulomas. Additionally, transcripts to IL-17 were reported within granulomatous lesions in murine models, indicating the participation of Th17 immunity in PCM granulomas (Pagliari et al., 2011; De Castro et al., 2013). The resistance to disease mediated by IFN-γ and tumor necrosis factor-alpha (TNF-α) produced by Th1 immunity, results in activated macrophages that characterize the asymptomatic individuals. The severe acute form of the disease, also called the “juvenile form”, has a predominance of a Th2/Th9 profile with a strong humoral response and high production of antibodies. Severe cases of the chronic form of PCM can also present elevated IL-10 and IL-4 production due to prevalent Th2 immunity. Furthermore, the predominance of a Th17 immune response concomitant with a robust participation of Th1 immunity mediated has also been described in the inflammatory response that characterizes the chronic form of the disease (Borges et al., 2023).

The immunoregulatory mechanisms that control resistance to PCM are multifaceted and not yet completely solved. Previous studies revealed that host immunity is tightly regulated by several suppressive mechanisms mediated by tolerogenic plasmacytoid dendritic cells, the enzyme 2,3 indoleamine dioxygenase (IDO-1), the transcription factor Aryl Hydrocarbon Receptor (AhR) regulatory T-cells (Tregs), and MDSCs (Felonato et al., 2012; Pina et al., 2013; Araújo et al., 2016; de Araújo et al., 2017a; de Araújo et al., 2017b; Preite et al., 2018; de Araújo et al., 2020; de Araújo et al., 2021). In a previous study, we demonstrated that the increased influx of MDSCs into the lungs of *P. brasiliensis-*infected mice was linked to more severe disease and impaired Th1 and Th17 protective responses. Besides, partial reduction of MDSC using anti-Gr1 antibody led to a robust Th1/Th17 response, resulting in regressive disease as revealed by reduced fungal burden on target organs, diminishing lung pathology, and diminished mortality ratio as compared with control IgG2b-treated mice. The suppressive activity of MDSCs on CD4^+^ and CD8^+^ T-lymphocytes and Th1/Th17 cells was also demonstrated *in vitro* using coculture experiments. Conversely, the adoptive transfer of MDSCs to *P. brasiliensi*s-infected mice resulted in a more severe disease (Preite et al., 2023).

Dectin-1, TLR2, and TLR4 are pattern recognition receptors (PRRs) classically recognized as important sensors for the pathogen-associated molecular patterns (PAMPs) of fungi (Romani, 2011). Dectin-1 is a specific receptor for β-glucans, which are found in abundance in the cell walls of fungi (Brown et al., 2003). The involvement of the Dectin-1 receptor has already been extensively studied in murine models of PCM (Calich et al., 2008; Loures et al., 2014; Loures et al., 2015; Preite et al., 2018). The importance of TLR2 and TLR4 has also been investigated in murine (Loures et al., 2009; Loures et al., 2010) and human PCM (Bonfim et al., 2009) by our research group. The recruitment of MDSCs as well as the production of immunosuppressive molecules by these cells has already been observed in different clinical contexts (Ray et al., 2013; Karumuthil-Melethil et al., 2014; Rieber et al., 2015; Zhai et al., 2017; Bahraoui et al., 2020; Oliveira et al., 2021). Infection by the fungi *Aspergillus fumigatus* and *Candida albicans* promotes the recruitment of MDSCs, whose mechanism of action depends on Dectin-1 signaling, leading to the production of reactive oxygen species with concomitant production of IL-1β (Rieber et al., 2015). In addition, monocytic MDSCs can be induced by TLR2 and TLR4 cognates derived from infectious agents such as hepatitis C virus (HCV) and *Staphylococcus aureus* (Zhai et al., 2017). Of note, *S. aureus* has TLR2 agonists that can induce the differentiation of MDSCs from monocytes, which leads to the accumulation of these suppressor cells in skin lesions (Skabytska et al., 2014; Dorhoi and Du Plessis, 2017). In addition, we have recently reported that IDO-1 expression by MDSCs is important for the control of T-cell proliferation and is partially dependent on Dectin-1, TLR2, and TLR4 signaling during *P. brasiliensis* infection in mice (Karumuthil-Melethil et al., 2014). Considering these previous findings, we aimed to investigate the influence of Dectin-1, TLR2, and TLR4 from MDSCs on the expression of PD-L1, IL-10, and NO suppressive molecules by MDSCs. Besides, we investigated the importance of these receptors for the suppressive activity of MDSCs on T-activated cells using coculture experiments.

## 2 Methods

### 2.1 Ethics statement

The experiments were performed in strict accordance with the Brazilian Federal Law 11,794 establishing procedures for the scientific use of animals and the State Law establishing the Animal Protection Code of the State of São Paulo. Also, the experiments were performed following the ARRIVE guidelines. All efforts were made to minimize animal suffering. The procedures were approved by the Ethics Committee on Animal Experiments of the Federal University of São Paulo-UNIFESP (Protocol N° 2135170220).

### 2.2 Mice

Eight- to 12-week-old male C57BL/6J WT, TLR2KO, and TLR4KO mice were bred as specific pathogen-free mice at the Center for the Development of Experimental Models for Biology and Medicine, Federal University of São Paulo – CEDEME-UNIFESP, and kept in the Facility of the Institute of Science and Technology of the Federal University of São Paulo – ICT-UNIFESP in São José dos Campos. Also, 8- to 12-week-old male C57BL/6 (backcrossed for at least nine generations) Dectin-1 KO and WT mice were obtained from the specific pathogen-free Isogenic Breeding Unit of the Department of Immunology, Institute of Biomedical Sciences, University of São Paulo, and kept in the Facility of ICT-UNIFESP. In agreement with ethical recommendations regarding the use and demand of genetically modified animals, we used cells from a few wild-type animals to differentiate MDSCs. We then used mAbs against the PRRs of interest as an exploratory study into the possible effects of the non-availability of these PRRs in these suppressor cells. Without preliminary results (replicated or not with KO cells or animals), the use of genetically modified animals at first would not be ethically acceptable to the committees.

### 2.3 Fungal strain and mice infection

Virulent *P. brasiliensis* 18 (Pb18 isolate) yeast cells were maintained by weekly cultivation in Fava Netto culture medium at 37°C and used on days 7–8 of culture. The viability of yeasts, which was always higher than 95%, was determined using Janus Green B vital dye (Merck). Mice were anesthetized and subjected to intratracheally (it.) infection as previously described (Cano et al., 1995). In brief, after intraperitoneal (ip.) injection of ketamine (90 mg/kg) and xylazine (10 mg/kg), animals were infected with 1 × 10^6^ yeast cells in 50 μL phosphate-buffered saline (PBS) by surgical (it.) inoculation, which allowed direct dispensing of the fungal cells into the lungs.

### 2.4 Flow cytometry

Cell suspensions were added to 96-well round-bottoms plates, and Fc receptors were blocked using 10 ng/mL Fc block (eBiosciences) for 10 min at 4°C. Plates were washed twice with FACs Buffer (BioLegend). Afterward, 25 μL of a mixture containing 1% of each fluorochrome-conjugated antibody used for M-MDSC and PMN-MDSC identification was added per well and incubated for 25 min at 4°C. The conjugated antibodies used for MDSC identification were Live/Dead-BV510, CD45-BV605, CD11b-APCCy7, Ly6C-APC R700, and Ly6G-BV421. The **Supplementary Figures 1A–E** shows the gating strategy for MDSC identification. We also evaluated the surface expression of PD-L1 (CD274) using anti-mouse PD-L1 PECy7 (Biolegend) (**Supplementary Figure 1F**) and the intracellular expression of IL-10 and nitrotyrosine in MDSCs. For intracellular cell staining, cells were treated with fixation/permeabilization buffer (BD Biosciences) for 20 min at 4°C. Then, cells were washed and submitted to a 25 μL mix of a FACs buffer containing 2% of the anti-IL-10 PerCP (Biolegend) and anti-nitrotyrosine FITC. For *in vitro* experiments, a total of 50,000 events were acquired while for *in vivo* experiments 100,000 events were acquired, both using the FACSuite software (BD Biosciences) and a BD FACS Lyric equipment. Cell population analysis was performed using the FlowJo software (Tree Star).

### 2.5 *In vitro* generation of MDSCs from mice bone-marrow

Bone-marrow-derived MDSCs (BM-MDSCs) were generated from naïve C57BL/6 WT, Dectin-1KO, TLR2KO, and TLR4KO mice as previously described (Kurkó et al., 2014; Kaminski et al., 2023). Mice were euthanized with intraperitoneal (ip.) injections of ketamine (270 mg/kg) and xylazine (30 mg/kg). Then, BM cells were flushed out from the femurs and tibias of mice with a 1 mL syringe and Dulbecco’s Modified Eagle Medium (DMEM, Sigma-Aldrich) supplemented with 3% fetal bovine serum (FBS). For red blood cell (RBC) lysis, RBC lysis buffer (BioLegend) was used for 4 min. Cells were then washed with DMEM, and 7 × 10^5^ white blood cells per mL were seeded in cell culture bottles with DMEM supplemented with 10% FBS, recombinant murine IL-6, and granulocyte-monocyte colony-stimulating factor (GM-CSF), both at 10 ng/mL (Biolegend) for MDSCs stimulation. BM cells were cultured for three days at 37°C in a 5% CO_2_ chamber. BM-MDSCs were positively isolated from other myeloid cell populations using the MDSC-isolation kit (Miltenyi), following the manufacturer’s instructions. Concerning the yeast-cells ratio used for *in vitro* challenge of MDSCs with *P. brasiliensis*, in our previous work investigating MDSCs in murine PCM we observed that an intratracheal infection of mice with 1 million *P. brasiliensis* yeasts resulted in the recovery of approximately 8 million MDSCs (comprising 3 million monocytic and 5 million polymorphonuclear cells) from the lungs after 72 hours, representing an estimated ratio of 1:8 (Preite et al., 2023). It is important to mention that, during *in vivo* infection (or natural infection), the presence of the fungus in the host’s tissues triggers the entire inflammatory response, including MDSC recruitment. Our pilot experiments on the *in vitro* fungal challenge started with yeast-to-MDSC ratios of 1:8 and 1:10. However, at a 1:10 ratio, cell death occurred, preventing co-culture with lymphocytes and subsequent flow cytometry analysis. To improve cell viability, we increased the proportion of cells, as smaller proportions yielded few live MDSCs for flow cytometric analysis involving intracellular cell staining (Kaminski et al., 2023).

### 2.6 Blockade of Dectin-1, TLR2, and TLR4 of BM-MDSCs by mAbs

BM-MDSCs obtained from C57BL/6 WT mice were treated or not with 10μg/mL of anti-Dectin-1 (Thermo Fisher), anti-TLR2, or anti-TLR4 (both from Invitrogen). For each treatment, 2 × 10^5^ BM-MDSCs were seeded in a 96-well round-bottom plate. A monoclonal IgG2b (BioxCell) was used as an isotype control.

### 2.7 Immunosuppression of BM-MDSCs on T-lymphocytes

Single-cell suspensions were generated from the spleens of naïve C57BL/6 WT mice. After RBC lysis, T-cell populations were isolated from splenocytes using the Pan T-Cell Isolation Kit (Miltenyi) according to the manufacturer’s instructions. To analyze the proliferation of T-lymphocytes, cells were stained with carboxyfluorescein succinimidyl ester (CellTrace™ CFSE Cell Proliferation Kit, Invitrogen) according to the manufacturer’s instructions. Subsequently, 1 × 10^6^ T-cells were activated with 1 μg/mL anti-CD3 and anti-CD28 monoclonal antibodies (BioLegend) and cultured at 37°C and 5% CO_2_ for four days in the presence or absence of 1 × 10^5^ BM-MDSCs per well in a 96-well round-bottom plate. Of note, BM-MDSCs were previously challenged or not with 4 × 10^3^ P*. brasiliensis* yeasts. T-cell proliferation was defined according to the CFSE dilution and assessed by flow cytometry as previously shown (**Supplementary Figure 2**) (Mannering et al., 2003; Kaminski et al., 2023).

### 2.8 Lung infiltrating MDSCs in WT, Dectin-1KO, TLR2KO, and TLR4KO infected mice

Lungs were collected from C57BL/6 WT, Dectin-1KO, TLR2KO, and TLR4KO *P. brasiliensis*-infected mice 72 h, two weeks, and eight weeks post-infection. Lung tissue was enzymatically digested in RPMI medium with 10% FBS containing 2 mg/mL of collagenase (Sigma-Aldrich) at 37°C for 40 min and 120 rpm in a shaker incubator. Lung leukocyte suspensions were obtained as previously described (Loures et al., 2014) and cells were subjected to cell staining as described above.

### 2.9 Statistical analysis

Statistical analysis was performed using GraphPad Prism, version 8. Comparisons of two Groups, means and ± standard errors were analyzed by unpaired Student’s *t*-test. For comparisons with more than two groups, significance was determined using One-Way ANOVA with the Tukey test.

## 3 Results

### 3.1 Dectin-1 absence decreased the number of lung-infiltrating M-MDSCs PD-L1^+^ after 72 h of infection

**Figure 1:**
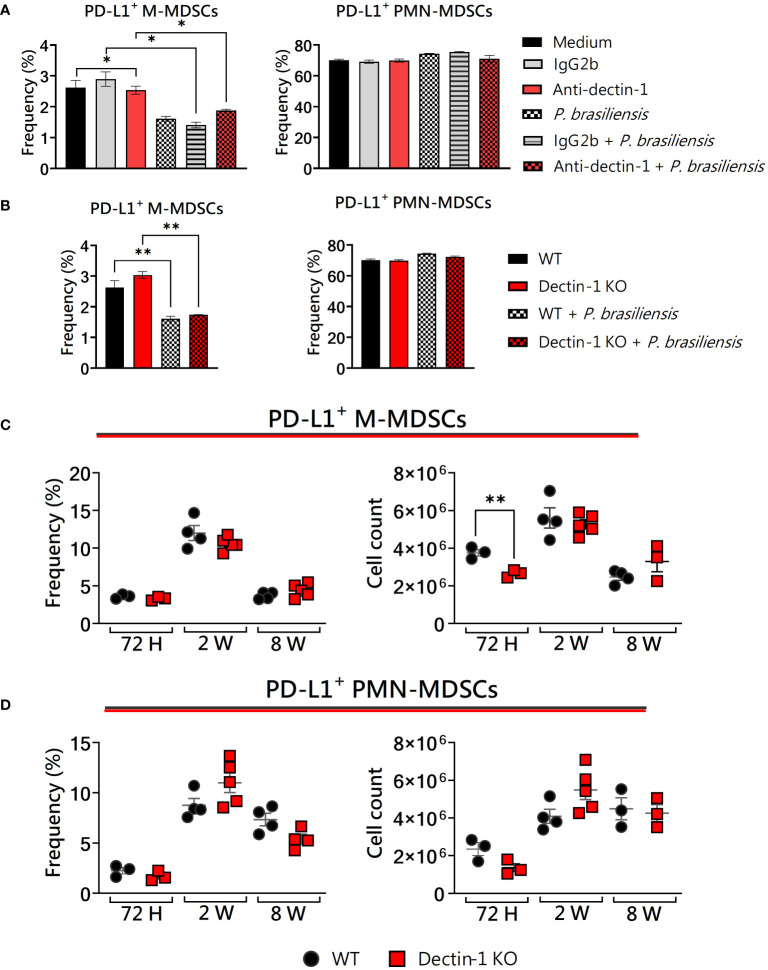
The role of Dectin-1 signaling in PD-L1 expression by MDSCs. MDSCs were generated *in vitro* from C57BL/6 wild-type (WT) mice and cultured in 96-well plates (2 × 10^5^ cells per well) and challenged with *P. brasiliensis* yeasts at a 1:25 ratio (yeast: MDSCs) overnight or not (control). In addition, some wells were treated with 10 µg/mL of anti-Dectin-1 or IgG2b for 2 h before the fungal challenge. The same fungal challenge was used in MDSCs generated from WT and Dectin-1KO mice. **(A)** The frequencies of PD-L1+ M- and PMN-MDSCs following treatment with anti-Dectin-1 or IgG2b control. **(B)** The frequencies of PD-L1+ M- and PMN-MDSCs generated *in vitro* from WT and Dectin-1 KO mice. Differences between groups were analyzed by analysis of variance (ANOVA) followed by the Tukey test. The data represent three independent experiments (N = 5 wells/treatment). Results were considered significant at *p < 0.05. **p < 0.01. **(C, D)** WT and Dectin-1 KO mice were intratracheally infected with 1 × 10^6^ *P. brasiliensis* yeasts. Lungs were collected after 72 h, two weeks, and eight weeks of infection. Adequate antibodies conjugated to fluorochromes were used to characterize MDSC subpopulations, as shown in **Supplementary Figure 1**. The frequency and total cell count of lung-infiltrating M-MDSCs **(C)** and PMN-MDSCs **(D)** positive to PD-L1 72 h, two weeks, and eight weeks post-infection were determined by comparing WT with Dectin-1KO. The data represent three independent experiments with 3–5 mice each. For comparisons between the two groups, the mean ± SEM was obtained and analyzed by the unpaired Student’s t-test. Differences were considered significant at *p < 0.05.**p < 0.01.

We have used three different approaches: cells from wild-type mice and receptor blockade with mAbs, bone marrow extraction from genetically modified (KOs) animals and subsequently *in vitro* generation of MDSCs, and finally, *in vivo* infection of WT and KO animals at different times of infection to confirm or not our findings obtained *in vitro*. We previously demonstrated that Dectin-1 deficiency influences the production of IDO-1, an important immunosuppressive mechanism of MDSCs in PCM (Kaminski et al., 2023; Preite et al., 2023). Here, we tested whether Dectin-1 could be involved in other immunosuppressive mechanisms of MDSCs, such as PD-L1 expression. The expression of PD-L1 in MDSCs was not affected by anti-Dectin-1 treatment or Dectin-1 absence *in vitro* (**Figures 1A, B**). Since the experiments in triplicate with mAbs showed some significant differences between the groups, we further investigated the effect in deficient animals for the genes coding for the molecules of interest. However, we could observe a reduced total number of PD-L1^+^ M-MDSCs after 72h of infection (**Figure 1C**), which could be a direct effect of the reduced number of M-MDSCs recruited to lungs 72 h post-infection (Kaminski et al., 2023). Interestingly, it has been shown that the expression of PD-L1 on murine and human neutrophils was upregulated upon the engagement of Dectin-1 in *C. albicans* yeast infection (Yu et al., 2022). However, our results regarding PD-L1^+^ PMN-MDSCs were similar between WT and Dectin-1KO mice (**Figure 1D**). These results suggest that different fungi may elicit distinct mechanisms in different immune cells, especially considering cells in different stages of differentiation, such as MDSCs.

### 3.2 Dectin-1 deficiency affected IL-10 production by MDSCs *in vitro* and *in vivo* after *P. brasiliensis* infection

**Figure 2:**
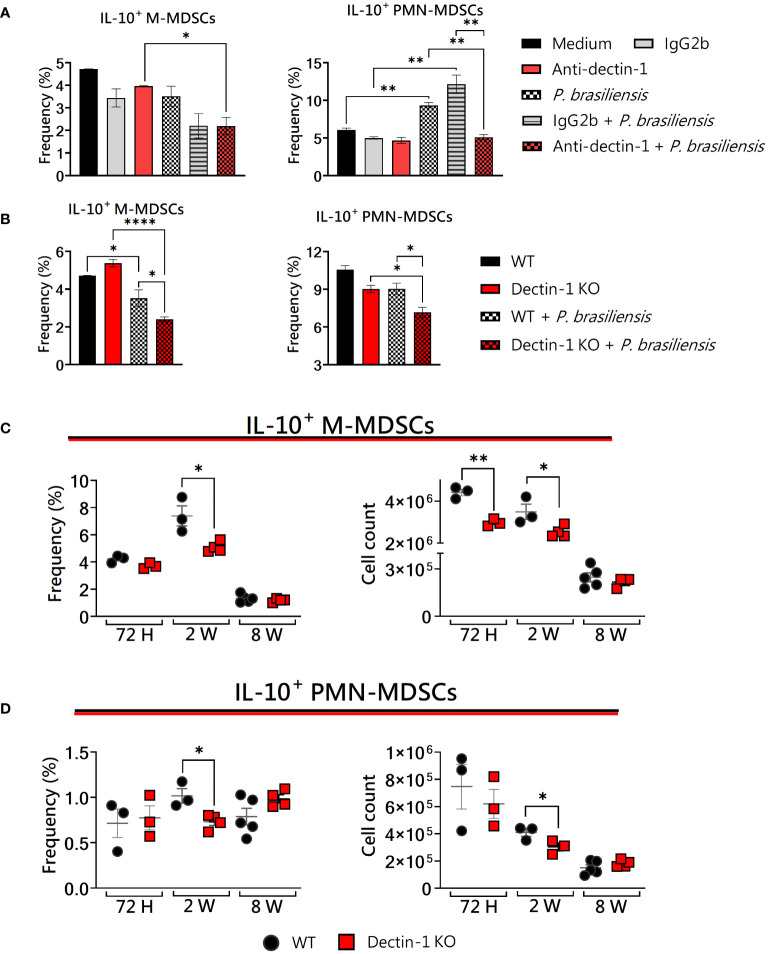
The role of Dectin-1 signaling in IL-10 production by MDSCs. MDSCs were generated *in vitro* from C57BL/6 wild-type (WT) mice and cultured in 96-well plates (2 × 10^5^ cells per well) and challenged with *P. brasiliensis* yeasts at a 1:25 ratio (yeast: MDSCs) overnight or not (control). In addition, some wells were treated with 10 µg/mL of anti-Dectin-1 or IgG2b for 2 h before the fungal challenge. The same fungal challenge was used in MDSCs generated from WT and Dectin-1 KO mice. **(A)** The frequencies of IL-10+ M- and PMN-MDSCs following treatment with anti-Dectin-1 or IgG2b control. **(B)** The frequencies of IL-10+ M- and PMN-MDSCs generated *in vitro* from WT and Dectin-1 KO mice. Differences between groups were analyzed by analysis of variance (ANOVA) followed by the Tukey test. The data represent three independent experiments (N = 5 wells/treatment). Results were considered significant at *p < 0.05. **p < 0.01 and ****p < 0.0001. **(C, D)** WT and Dectin-1 KO mice were intratracheally infected with 1 × 10^6^ *P. brasiliensis* yeasts. Lungs were collected after 72 h, two weeks, and eight weeks of infection. Adequate antibodies conjugated to fluorochromes were used to characterize MDSC subpopulations, as shown in **Supplementary Figure 1**. The frequency and total cell count of lung-infiltrating M-MDSCs **(C)** and PMN-MDSCs **(D)** positive for IL-10 72 h, two weeks, and eight weeks post-infection were determined by comparing WT with Dectin-1 KO. The data represent three independent experiments with 3–5 mice each. For comparisons between the two groups, the mean ± SEM was obtained and analyzed by the unpaired Student’s t-test. Differences were considered significant at *p < 0.05.**p < 0.01. Replicates of the *in vivo* experiments are shown in **Supplementary Figure 3**.

IL-10 production is another suppressive molecule used by MDSC activity during *P. brasiliensis* infection (Preite et al., 2023). Of note, it has been shown that Dectin-1 regulates IL-10 production via mitogen- and stress-activated protein kinase (MSK1/2) and cyclic AMP-response element-binding protein 1 (CREB), thus promoting the induction of regulatory macrophage markers after zymosan stimulation (Elcombe et al., 2013). As can be seen in **Figure 2A**, anti-Dectin-1 treatment diminished the ability of M- and PMN-MDSCs to produce IL-10 *in vitro* after yeast challenge, in addition to Dectin-1KO MDSCs that also presented impaired IL-10 expression *in vitro* (**Figure 2B**). *In vivo*, reduced IL-10^+^ M-MDSCs were observed in the lungs of Dectin-1KO mice after 72 h and 2 weeks of infection (**Figure 2C**), while PMN-MDSCs presented impairments in IL-10 expression 2 weeks post-infection (**Figure 2D**). Together, our results reinforce the important role of IL-10 as a potent immunosuppressive factor elicited by the activation of C-type lectin receptors by fungal pathogens.

### 3.3 Dectin-1 absence reduced the production of nitrotyrosine by MDSCs *in vitro* and *in vivo* after *P. brasiliensis* infection

**Figure 3:**
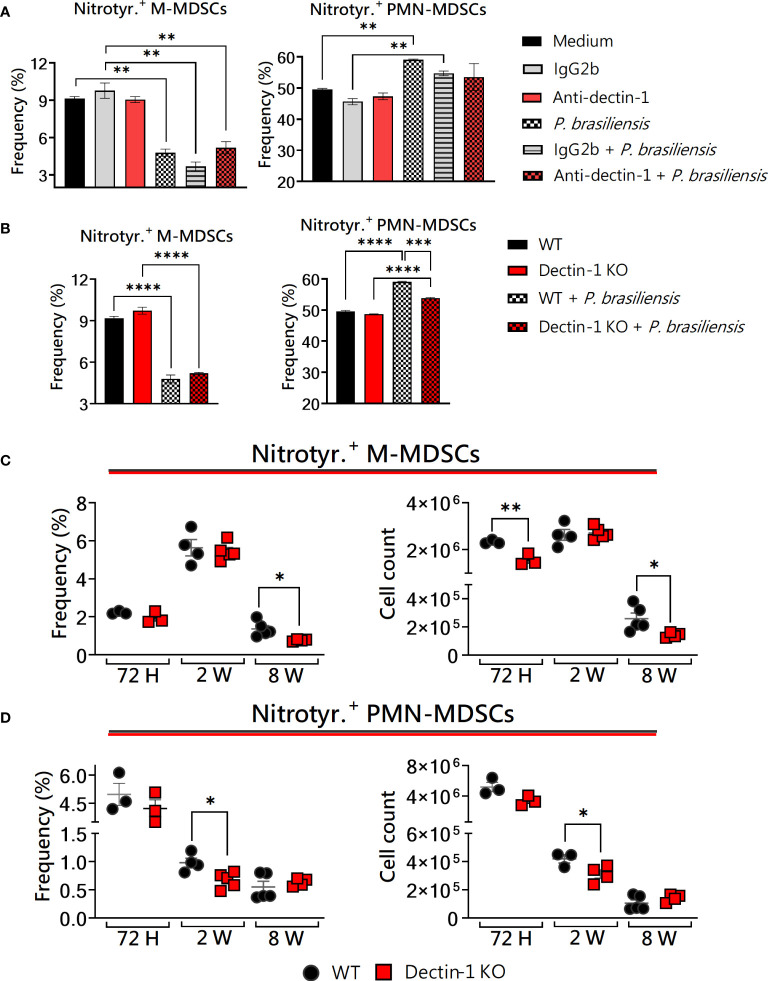
The role of Dectin-1 signaling in the generation of nitrotyrosine by MDSCs. MDSCs were generated *in vitro* from C57BL/6 wild-type (WT) mice and cultured in 96-well plates (2 × 10^5^ cells per well) and challenged with *P. brasiliensis* yeasts at a 1:25 ratio (yeast: MDSCs) overnight or not. In addition, some wells were treated with 10 µg/mL of anti-Dectin-1 or IgG2b for 2 h before the fungal challenge. The same fungal challenge was used in MDSCs generated from WT and Dectin-1 KO mice. **(A)** The frequencies of Nitrotyrosine+ M- and PMN-MDSCs following treatment with anti-Dectin-1 or IgG2b control. **(B)** The frequencies of nitrotyrosine+ M- and PMN-MDSCs generated *in vitro* from WT and Dectin-1KO mice. Differences between groups were analyzed by analysis of variance (ANOVA) followed by the Tukey test. The data represent three independent experiments (N = 5 wells/treatment). Results were considered significant at *p* < 0.05. **p < 0.01 and ****p < 0.0001. **(C, D)** WT and Dectin-1KO mice were intratracheally infected with 1 × 10^6^ *P. brasiliensis* yeasts. Lungs were collected after 72 h, two weeks, and eight weeks of infection. Adequate antibodies conjugated to fluorochromes were used to characterize MDSC subpopulations, as shown in **Supplementary Figure 1**. The frequency and total cell count of lung-infiltrating M-MDSCs **(C)** and PMN-MDSCs **(D)** positive for nitrotyrosine 72 h, two weeks, and eight weeks post-infection were determined by comparing WT with Dectin-1 KO. The data represent three independent experiments with 3–5 mice each. For comparisons between the two groups, the mean ± SEM was obtained and analyzed by the unpaired Student’s t-test. Differences were considered significant at *p < 0.05. **p < 0.01. Replicates of the *in vivo* experiments are shown in **Supplementary Figure 4**.

Peroxynitrite is the product of the reaction of nitric oxide (NO) and superoxide radicals, being a short-lived reactive peroxide and a good oxidant. Radicals derived from peroxynitrite act by promoting the nitration of proteins. A recognized protein modification left by peroxynitrite *in vitro* and *in vivo* is the formation of 3-nitrotyrosine (Radi, 2013). Of note, the immunosuppressive function of MDSCs has been related to the inducible nitric oxide synthase (iNOS) pathway, leading to NO synthesis and reactive oxygen species (ROS) generation (Ohl and Tenbrock, 2018). In this way, it is possible to evaluate peroxynitrite-producing MDSCs using an anti-nitrotyrosine antibody to residues in intracellular compartments. Here, we investigated the production of nitrotyrosine *in vitro* and *in vivo* in the context of Dectin-1 deficiency and *P. brasiliensis* yeast challenge. *In vitro*, no differences were observed regarding monocytic MDSCs (**Figures 3A, B**, left panels) when both WT and KO cells were challenged with *P. brasiliensis*. Additionally, PMN-MDSCs were unable to increase nitrotyrosine production after fungal infection after blocking Dectin-1 with a monoclonal antibody (**Figure 3A**, right panel). The production of nitrotyrosine by Dectin-1KO PMN-MDSCs was smaller than that of WT PMN-MDSCs (**Figure 3B**, right panel). The production of nitrotyrosine was also investigated in lung-infiltrating MDSCs from WT and Dectin-1KO animals. A lower frequency of nitrotyrosine^+^ M-MDSCs was observed after 8 weeks of infection in Dectin-1KO mice (**Figure 3C**, left panel) compared to WT counterparts. Besides, M-MDSCs from Dectin-1KO animals had a lower total number of nitrotyrosine-producing cells 72 hours and 8 weeks post-infection (**Figure 3C**, right panel). Interestingly, concerning lung infiltrating nitrotyrosine-producing PMN-MDSCs, lower frequency and number of these cells were observed in Dectin-1KO animals 2 weeks after infection (**Figure 3D**) compared to WT animals. Given the discrepancies observed in nitrotyrosine production between *in vitro* experiments with mAbs and Dectin-KO cells, it is important to emphasize the significance of *in vivo* experiments for elucidating the role of Dectin-1 in MDSCs activity. Considering this, **Supplementary Figure 3** presents the replicates of the *in vivo* experiments shown in **Figures 3C, D**.

### 3.4 *In vivo*, TLR2 absence affected the expression of PD-L1 by PMN-MDSCs 72 hours and 2 weeks post-infection

**Figure 4:**
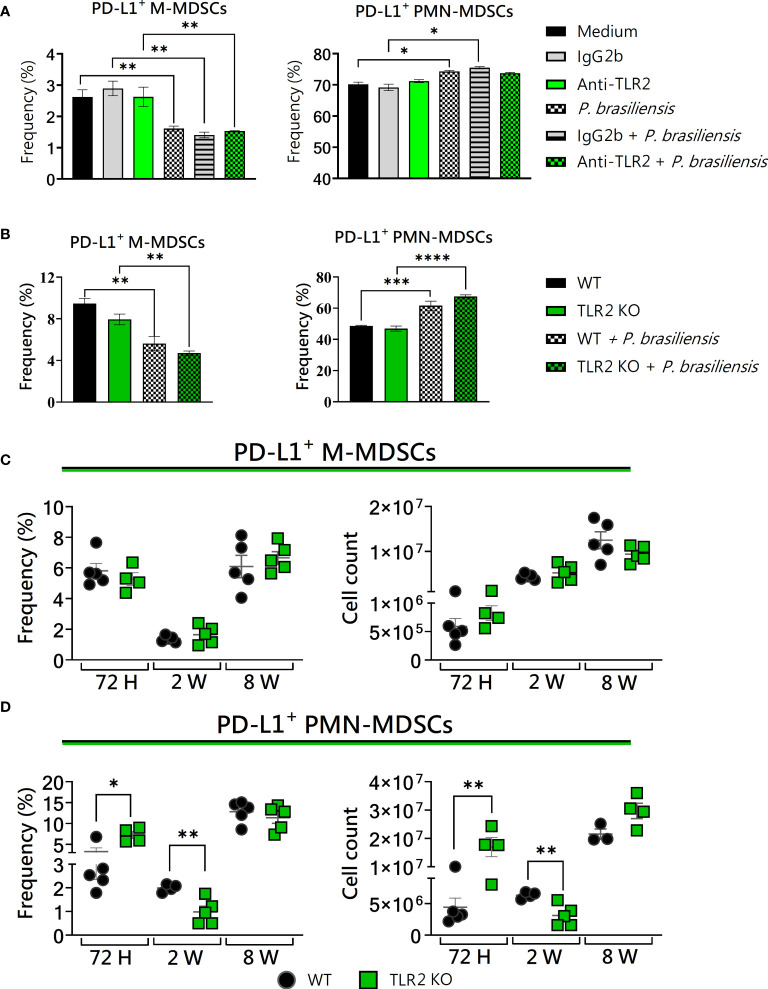
The role of TLR2 signaling in PD-L1 expression by MDSCs. MDSCs were generated *in vitro* from C57BL/6 wild-type (WT) mice and cultured in 96-well plates (2 × 10^5^ cells per well) and challenged with *P. brasiliensis* yeasts at a 1:25 ratio (yeast: MDSCs) overnight or not (control). In addition, some wells were treated with 10 µg/mL of anti-TLR2 or IgG2b for 2 h before the fungal challenge. The same fungal challenge was used in MDSCs generated from WT and TLR2KO mice. **(A)** The frequencies of PD-L1+ M- and PMN-MDSCs following treatment with anti-TLR2 or IgG2b control. **(B)** The frequencies of PD-L1+ M- and PMN-MDSCs generated *in vitro* from WT and TLR2KO mice. Differences between groups were analyzed by analysis of variance (ANOVA) followed by the Tukey test. The data represent three independent experiments (N = 5 wells/treatment). Results were considered significant at *p < 0.05. **p < 0.01. **(C, D)** WT and TLR2KO mice were intratracheally infected with 1 × 10^6^ *P. brasiliensis* yeasts. Lungs were collected after 72 h, two weeks, and eight weeks of infection. Adequate antibodies conjugated to fluorochromes were used to characterize MDSC subpopulations, as shown in **Supplementary Figure 1**. The frequency and total cell count of lung-infiltrating M-MDSCs **(C)** and PMN-MDSCs **(D)** positive to PD-L1 72 h, two weeks, and eight weeks post-infection were determined by comparing WT with TLR2KO. The data represent three independent experiments with 3–5 mice each. For comparisons between the two groups, the mean ± SEM was obtained and analyzed by the unpaired Student’s t-test. Differences were considered significant at *p < 0.05. **p < 0.01, ***p < 0.001, and ****p < 0.0001. Replicates of the *in vivo* experiments are shown in **Supplementary Figure 5**.

In our investigations into the role of TLR2 and TLR4 in the activity of MDSCs, we also employed three distinct approaches: the use of wild-type cells, receptor blockade, and validation of results using cells from receptor knockout mice and *in vivo* infection. The signaling through TLRs in MDSCs has been extensively investigated in various infectious diseases (Tacke et al., 2012; Skabytska et al., 2014; Pang et al., 2016; Ren et al., 2016; Dorhoi and Du Plessis, 2017; Zhai et al., 2017). To assess the influence of TLR2 on *in vitro*-generated MDSCs challenged with *P. brasiliensis* yeast, we used bone marrow cells from WT and TLR2KO mice. Administration of anti-TLR2 and the use of TLR2KO mice showed no alteration in PD-L1 expression in MDSCs *in vitro* (**Figures 4A, B**). Furthermore, *in vivo*, PD-L1 expression in M-MDSCs was unaffected (**Figure 4C**). However, when analyzing PMN-MDSCs from lung infiltrates of TLR2KO mice, we observed an increase in PD-L1 expression after 72 hours, followed by a reduction after two weeks of infection (**Figure 4D**). These data suggest little effect of TLR2 on PD-L1 expression by MDSCs, but a dual influence on PMN-MDSCs recruited to the site of infection during the acute phase of the disease.

### 3.5 Deficiency in TLR2 signaling altered the expression of IL-10 by MDSCs

**Figure 5:**
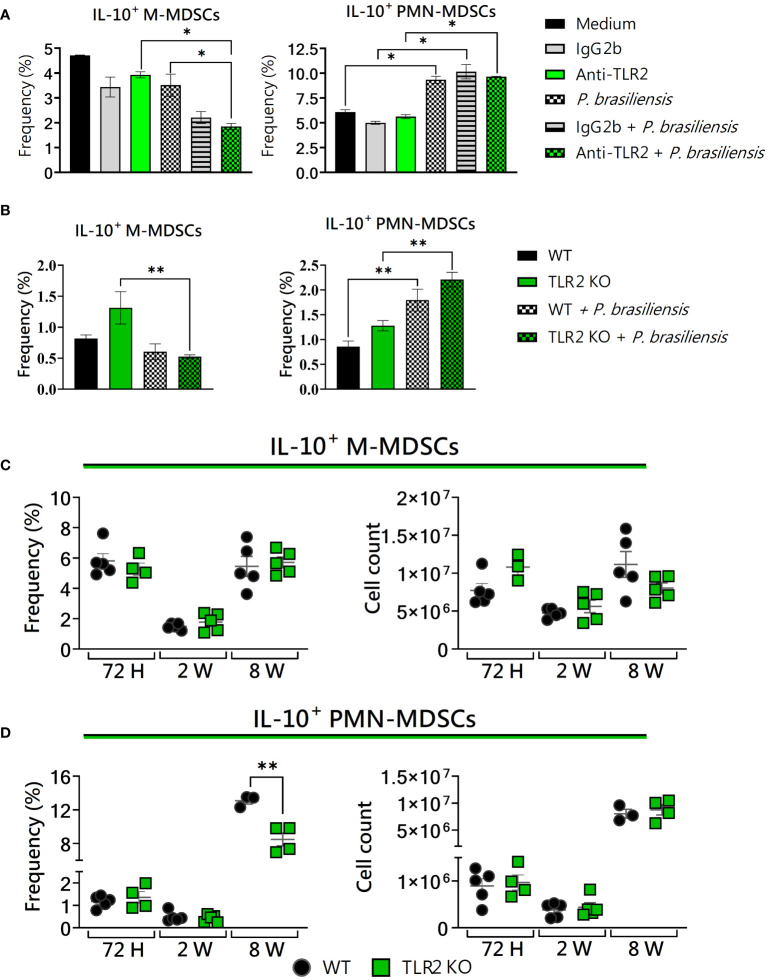
The role of TLR2 signaling in IL-10 production by MDSCs. MDSCs were generated *in vitro* from C57BL/6 wild-type (WT) mice and cultured in 96-well plates (2 × 10^5^ cells per well) and challenged with *P. brasiliensis* yeasts at a 1:25 ratio (yeast: MDSCs) overnight or not (control). In addition, some wells were treated with 10 µg/mL of anti-TLR2 or IgG2b for 2 h before the fungal challenge. The same fungal challenge was used in MDSCs generated from WT and TLR2KO mice. **(A)** The frequencies of IL-10+ M- and PMN-MDSCs following treatment with anti-TLR2 or IgG2b control. **(B)** The frequencies of IL-10+ M- and PMN-MDSCs generated *in vitro* from WT and TLR2KO mice. Differences between groups were analyzed by analysis of variance (ANOVA) followed by the Tukey test. The data represent three independent experiments (N = 5 wells/treatment). Results were considered significant at *p < 0.05. **p < 0.01. **(C, D)** WT and TLR2KO mice were intratracheally infected with 1 × 10^6^ *P. brasiliensis* yeasts. Lungs were collected after 72 h, two weeks, and eight weeks of infection. Adequate antibodies conjugated to fluorochromes were used to characterize MDSC subpopulations, as shown in **Supplementary Figure 1**. The frequency and total cell count of lung-infiltrating M-MDSCs **(C)** and PMN-MDSCs **(D)** positive to IL-10 72 h, two weeks, and eight weeks post-infection were determined by comparing WT with TLR2KO. The data represent three independent experiments with 3–5 mice each. For comparisons between the two groups, the mean ± SEM was obtained and analyzed by the unpaired Student’s t-test. Differences were considered significant at *p* < 0.05. **p < 0.01.

Previous studies have suggested that MDSCs are the main producers of IL-10 in tumors and infections (Huang et al., 2006; Ren et al., 2016). Then, we also evaluated if TLR2 deficiency would interfere with the IL-10 production by these suppressive cells in the context of *P. brasiliensis* infection. After treatment with anti-TLR2 and the use of TLR2KO cells, TLR2-deficient PMN-MDSCs showed no change in IL-10 expression compared to controls (**Figures 5A, B**). However, a reduced frequency of IL-10^+^ M-MDSCs was observed after anti-TLR2 treatment or in TLR2 absence after yeast challenge (**Figures 5A, B**). Under *in vivo* conditions, at 72 hours and 2 weeks post-infection, results regarding IL-10^+^ MDSCs were similar between WT and TLR2-deficient mice (**Figures 5C, D**). However, after 8 weeks of infection, TLR2KO mice presented a reduced frequency of IL-10+ PMN-MDSCs compared to WT mice (**Figure 5D**). These results indicate that TLR2 deficiency led to a reduction in the presence of MDSCs expressing IL-10 recruited to the lungs during *P. brasiliensis* infection in the chronic phase of the disease. Therefore, our results corroborate previous data from other studies that showed IL-10 as an important suppressive mechanism for MDSCs (Dorhoi and Du Plessis, 2017).

### 3.6 *In vivo*, TLR2 absence led to a reduction in MDSCs expressing nitrotyrosine

**Figure 6:**
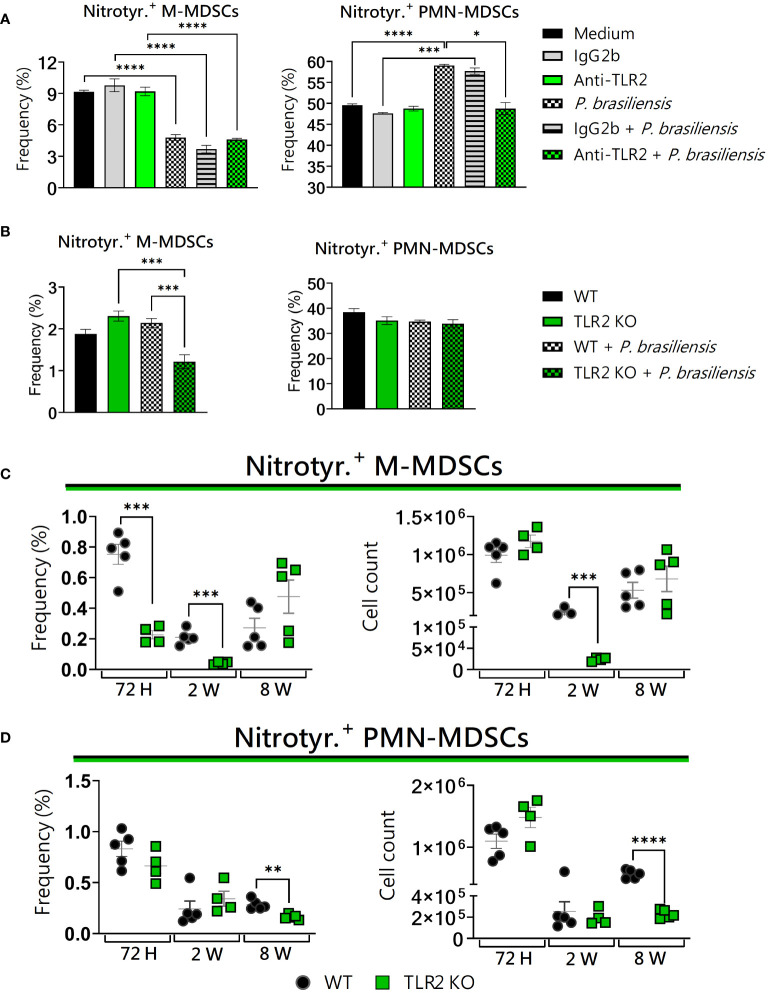
The role of TLR2 signaling in nitrotyrosine generation by MDSCs. MDSCs were generated *in vitro* from C57BL/6 wild-type (WT) mice and cultured in 96-well plates (2 × 10^5^ cells per well) and challenged or not with *P. brasiliensis* yeasts at a 1:25 ratio (yeast: MDSCs) overnight. In addition, some wells were treated with 10 µg/mL of anti-TLR2 or IgG2b for 2 h before the fungal challenge. The same fungal challenge was used in MDSCs generated from WT and TLR2KO mice. **(A)** The frequencies of nitrotyrosine+ M- and PMN-MDSCs following treatment with anti-TLR2 or IgG2b control. **(B)** The frequencies of nitrotyrosine+ M- and PMN-MDSCs generated *in vitro* from WT and TLR2KO mice. Differences between groups were analyzed by analysis of variance (ANOVA) followed by the Tukey test. The data represent three independent experiments (N = 5 wells/treatment). Results were considered significant at *p < 0.05. **p < 0.01. **(C, D)** WT and TLR2KO mice were intratracheally infected with 1 × 10^6^ *P. brasiliensis* yeasts. Lungs were collected after 72 h, two weeks, and eight weeks of infection. Adequate antibodies conjugated to fluorochromes were used to characterize MDSC subpopulations, as shown in **Supplementary Figure 1**. The frequency and total cell count of lung-infiltrating M-MDSCs **(C)** and PMN-MDSCs **(D)** positive to nitrotyrosine 72 h, two weeks, and eight weeks post-infection were determined by comparing WT with TLR2KO. The data represent three independent experiments with 3–5 mice each. For comparisons between the two groups, the mean ± SEM was obtained and analyzed by the unpaired Student’s t-test. Differences were considered significant at *p* < 0.05. **p < 0.01, ***p < 0.001, and ****p < 0.0001.

*In vitro*, both TLR2-blocked PMN-MDSCs as well as M-MDSCs TLR2KO presented alterations regarding nitrotyrosine expression (**Figures 6A, B**). In agreement, *in vivo*, nitrotyrosine^+^ M-MDSCs underwent a reduction 72 hours and 2 weeks post-infection, while PMN-MDSCs showed a reduction only at 8 weeks (**Figures 6C, D**). Taken together, our results indicate that the absence of TLR2 had a direct impact on the reduction of nitrotyrosine expression in MDSCs. In a recent study from our group, an increase in nitrotyrosine^+^ PMN-MDSCs was observed after 8 weeks of infection with *P. brasiliensis* (Preite et al., 2023).

### 3.7 TLR4 absence reduced the frequency of PD-L1^+^ PMN-MDSCs

**Figure 7:**
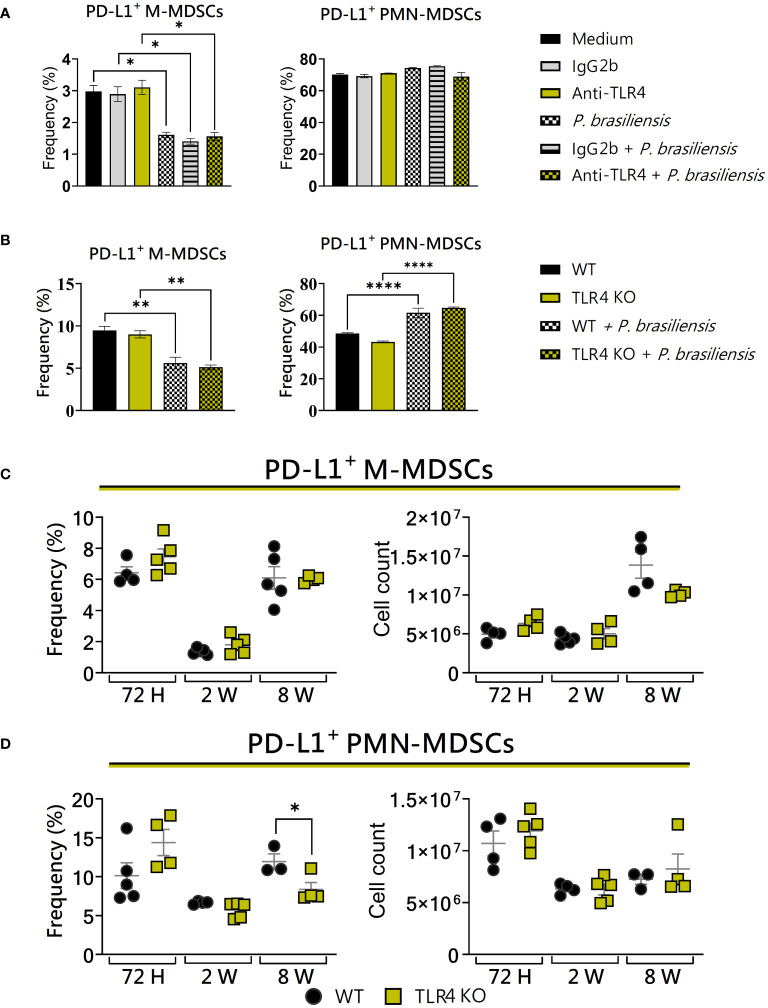
The role of TLR4 signaling in PD-L1 expression by MDSCs. MDSCs were generated *in vitro* from C57BL/6 wild-type (WT) mice and cultured in 96-well plates (2 × 10^5^ cells per well) and challenged or not with *P. brasiliensis* yeasts at a 1:25 ratio (yeast: MDSCs) overnight. In addition, some wells were treated with 10 µg/mL of anti-TLR4 or IgG2b for 2 h before the fungal challenge. The same fungal challenge was used in MDSCs generated from WT and TLR4KO mice. **(A)** The frequencies of PD-L1+ M- and PMN-MDSCs following treatment with anti-TLR4 or IgG2b control. **(B)** The frequencies of PD-L1+ M- and PMN-MDSCs generated *in vitro* from WT and TLR4KO mice. Differences between groups were analyzed by analysis of variance (ANOVA) followed by the Tukey test. The data represent three independent experiments (N = 5 wells/treatment). Results were considered significant at *p < 0.05. **p < 0.01 and ****p < 0.0001. **(C, D)** WT and TLR4KO mice were intratracheally infected with 1 × 10^6^ *P. brasiliensis* yeasts. Lungs were collected after 72h, two weeks, and eight weeks of infection. Adequate antibodies conjugated to fluorochromes were used to characterize MDSC subpopulations, as shown in **Supplementary Figure 1**. The frequency and total cell count of lung-infiltrating M-MDSCs **(C)** and PMN-MDSCs **(D)** positive to PD-L1 72 h, two weeks, and eight weeks post-infection were determined by comparing WT with TLR4KO. The data represent three independent experiments with 3–5 mice each. For comparisons between the two groups, the mean ± SEM was obtained and analyzed by the unpaired Student’s t-test. Differences were considered significant at *p < 0.05.

As the suppressive response mediated by MDSCs is highly dynamic and dependent on the microenvironment (Peñaloza et al., 2019), we decided to assess the impact of TLR4 blocking and deficiency on the expression of PD-L1 by MDSCs. *In vitro*, no differences were observed between WT and TLR4-blocked or TLR4KO MDSCs regarding the expression of PD-L1 (**Figures 7A, B**), as well as among PD-L1^+^ M-MDSCs in the lungs of WT and TLR4KO animals (**Figure 7C**). Only regarding PMN-MDSCs we could observe a slight decrease in PD-L1 expression in TLR4KO animals after 8 weeks of infection (**Figure 7D**). This indicates that TLR4 absence has little effect on the frequency of PD-L1^+^ MDSCs recruited to the lungs of *P. brasiliensis*-infected mice, with an effect detected only in polymorphonuclear populations.

### 3.8 TLR4 deficiency reduced the frequency of IL-10^+^ M-MDSCs

**Figure 8:**
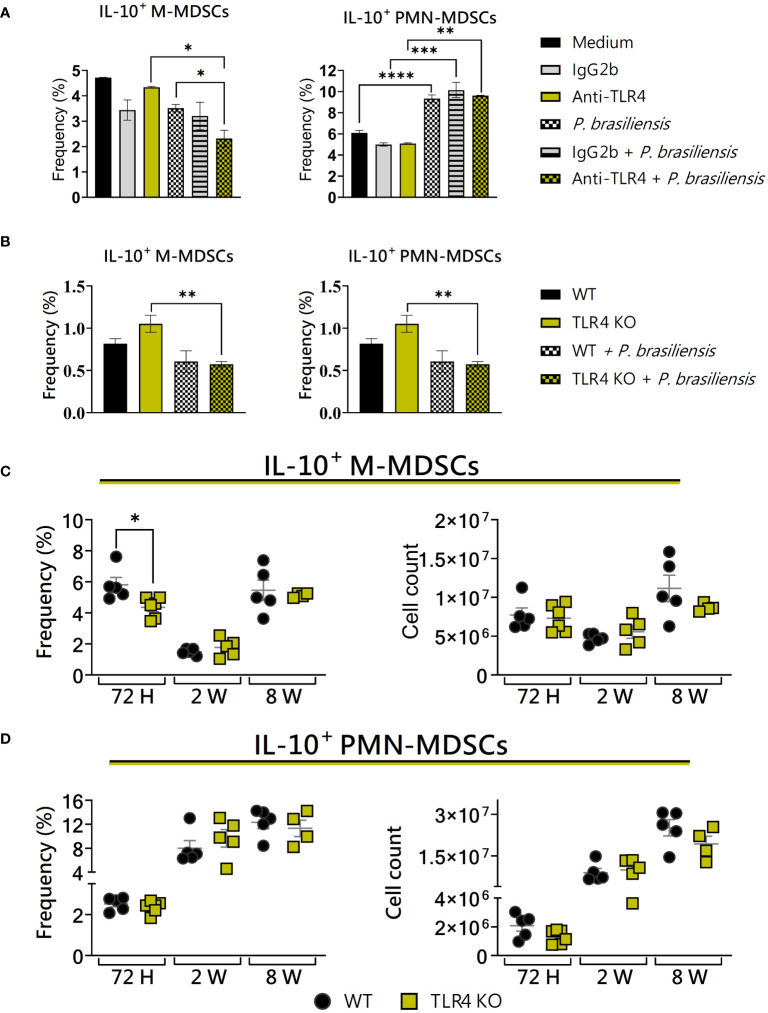
The role of TLR4 signaling in IL-10 production by MDSCs. MDSCs were generated *in vitro* from C57BL/6 wild-type (WT) mice and cultured in 96-well plates (2 × 10^5^ cells per well) and challenged or not with *P. brasiliensis* yeasts at a 1:25 ratio (yeast: MDSCs) overnight. In addition, some wells were treated with 10 µg/mL of anti-TLR4 or IgG2b for 2 h before the fungal challenge. The same fungal challenge was used in MDSCs generated from WT and TLR4KO mice. **(A)** The frequencies of IL-10+ M- and PMN-MDSCs following treatment with anti-TLR4 or IgG2b control. **(B)** The frequencies of IL-10+ M- and PMN-MDSCs generated *in vitro* from WT and TLR4KO mice. Differences between groups were analyzed by analysis of variance (ANOVA) followed by the Tukey test. The data represent three independent experiments (N = 5 wells/treatment). Results were considered significant at *p < 0.05. **p < 0.01, ***p < 0.001 and ****p < 0.0001. **(C, D)** WT and TLR4KO mice were intratracheally infected with 1 × 10^6^ *P. brasiliensis* yeasts. Lungs were collected after 72 h, two weeks, and eight weeks of infection. Adequate antibodies conjugated to fluorochromes were used to characterize MDSC subpopulations, as shown in **Supplementary Figure 1**. The frequency and total cell count of lung-infiltrating M-MDSCs **(C)** and PMN-MDSCs **(D)** positive to IL-10 72 h, two weeks, and eight weeks post-infection were determined by comparing WT with TLR4KO. The data represent three independent experiments with 3–5 mice each. For comparisons between the two groups, the mean ± SEM was obtained and analyzed by the unpaired Student’s t-test. Differences were considered significant at *p < 0.05.

Some studies, including Arora et al. (2010) (Arora et al., 2010) and Su et al. (2017) (Su et al., 2017), have identified the presence of TLR4^+^ MDSCs in infectious processes. In coculture experiments involving MDSCs and macrophages from BALB/c WT and TLR4KO mice, it was observed that MDSCs from WT animals presented an increased inflammation due to tumors and produced significantly more IL-10 as compared to controls (Bunt et al., 2009). Considering these, we have evaluated the influence of TLR4 on the expression of IL-10 by MDSCs in murine paracoccidioidomycosis. TLR4 deficiency *in vitro* resulted in a reduction in the frequency of IL-10^+^ M-MDSCs (**Figure 8A**), while no differences in IL-10 production were observed concerning PMN-MDSCs from WT and TLR4KO mice (**Figure 8B**).

Considering the expression of IL-10 by MDSCs recruited to the lungs of infected mice, the only difference observed between WT and TLR4KO animals was a lower frequency of IL-10^+^ M-MDSCs in TLR4-deficient mice after 72 hours of infection. There were no differences in the other time points studied (**Figure 8C**). Similarly, no difference was observed between the studied groups regarding the production of IL-10 by PMN-MDSCs (**Figure 8D**). Our results suggest that, *in vivo*, TLR4 deficiency leads to a reduction in IL-10^+^ M-MDSCs during the acute phase of the disease.

### 3.9 TLR4 deficiency reduced the expression of nitrotyrosine by PMN-MDSCs *in vitro*

**Figure 9:**
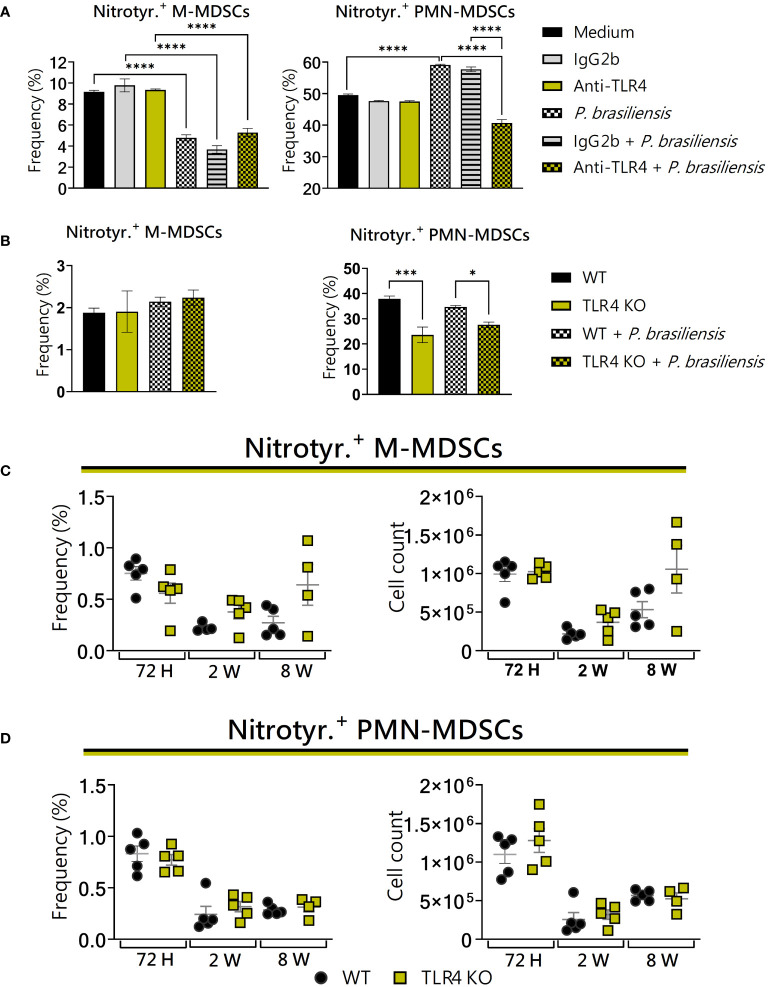
The role of TLR4 signaling in nitrotyrosine generation by MDSCs. MDSCs were generated *in vitro* from C57BL/6 wild-type (WT) mice and cultured in 96-well plates (2 × 10^5^ cells per well) and challenged or not with *P. brasiliensis* yeasts at a 1:25 ratio (yeast: MDSCs) overnight. In addition, some wells were treated with 10 µg/mL of anti-TLR4 or IgG2b for 2 h before the fungal challenge. The same fungal challenge was used in MDSCs generated from WT and TLR4KO mice. **(A)** The frequencies of nitrotyrosine+ M- and PMN-MDSCs following treatment with anti-TLR4 or IgG2b control. **(B)** The frequencies of nitrotyrosine+ M- and PMN-MDSCs generated *in vitro* from WT and TLR4KO mice. Differences between groups were analyzed by analysis of variance (ANOVA) followed by the Tukey test. The data represent three independent experiments (N = 5 wells/treatment). Results were considered significant at *p < 0.05. ***p < 0.001 and ****p < 0.0001. **(C, D)** WT and TLR4KO mice were intratracheally infected with 1 × 10^6^ *P. brasiliensis* yeasts. Lungs were collected after 72 h, two weeks, and eight weeks of infection. Adequate antibodies conjugated to fluorochromes were used to characterize MDSC subpopulations, as presented in **Supplementary Figure 1**. The frequency and total cell count of lung-infiltrating M-MDSCs **(C)** and PMN-MDSCs **(D)** positive to nitrotyrosine 72 h, two weeks, and eight weeks post-infection were determined by comparing WT with TLR4KO. The data represent three independent experiments with 3–5 mice each. For comparisons between the two groups, the mean ± SEM was obtained and analyzed by the unpaired Student’s t-test. Differences were considered significant at *p* < 0.05.

*In vitro*, TLR4 deficiency had no effect on the production of nitrotyrosine by M-MDSCs (**Figures 9A, B**, left panels). However, regarding PMN-MDSCs, we observed a decrease in nitrotyrosine production (**Figures 9A, B**, right panels). No difference between the groups was detected regarding the production of nitrotyrosine by MDSCs recruited to the lungs of WT and TLR4KO mice (**Figures 9C, D**). Thus, considering that the defects in nitrotyrosine production observed *in vitro* were not reproduced *in vivo*, the effects observed *in vitro* may be due to the absence of additional signaling present at the infectious site.

### 3.10 Dectin-1, TLR2 and TLR4 contribute to the suppressive capacity of MDSCs on T-cells

**Figure 10:**
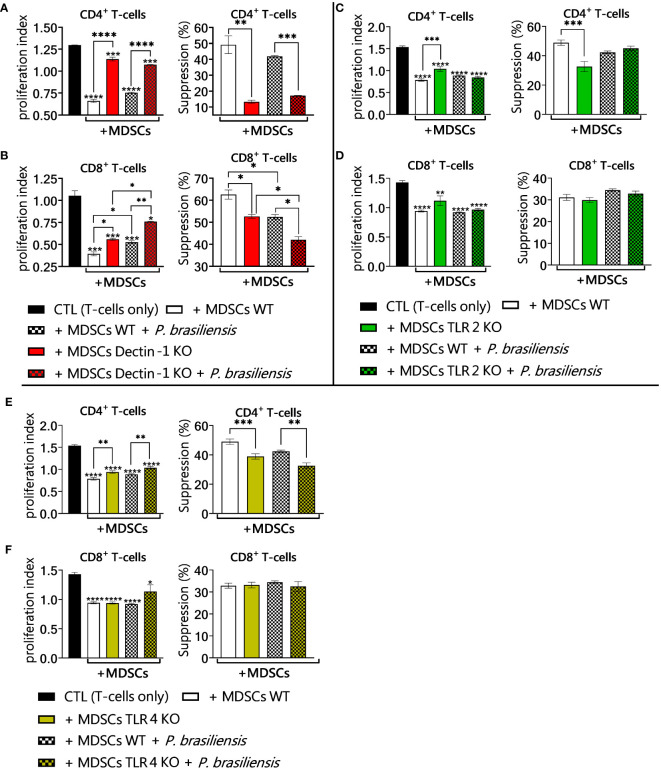
Suppression of T-cell proliferation by MDSCs. To evaluate the influence of MDSCs PRRs Dectin-1, TLR2, and TLR4 in suppressing T-cell proliferation, *in vitro* generated MDSCs were obtained from both wild-type (WT) and Dectin-1KO, TLR2KO and TLR4KO mice. Subsequently, these MDSCs were challenged with *P. brasiliensis* yeasts at a rate of 1:50 (yeast: MDSCs) and cocultured with 1 × 10^6^ CFSE-labeled T-cells per well in a 96-well round-bottom plate. T-cells were activated previously with 1 μg/mL of anti-CD3/CD28 antibodies. Following four days of coculture (ratio of 1:10 MDSC: T-cells), the frequency of CD4 and CD8 T-cells was characterized by flow cytometric analysis. The cell proliferation indices were obtained. The control T-cells (CTL) were cultured without contact with MDSCs. **(A, B)** The frequency and proliferation index of CD4+T-cells **(A)** and CD8+T-cells **(B)** in coculture with WT and Dectin-1KO MDSCs. **(C, D)** The frequency and proliferation index of CD4+T-cells **(C)** and CD8+T-cells **(D)** in coculture with WT and TLR2KO MDSCs. **(E, F)** The frequency and proliferation index of CD4+T-cells **(E)** and CD8+T-cells **(F)** in coculture with WT and TLR4KO MDSCs. The data represent three independent experiments for each PRR approached (N = 5 wells/group). Differences between treatments were analyzed by analysis of variance (ANOVA) followed by the Tukey test. Results were considered significant at *p < 0.05; **p < 0.01; ***p < 0.001, and ****p < 0.0001.

To verify whether the changes in the production of immunosuppressive molecules observed in the defective signaling of Dectin-1, TLR2, and TLR4 in MDSCs could play a role in the suppressive capacity of these cells on activated T lymphocytes, coculture experiments were performed. T lymphocytes were isolated from the spleens of WT and Dectin-1 KO, TLR2KO, and TLR4KO mice. Lymphocytes were activated and cocultured with MDSCs exposed or not to *P. brasiliensis* yeast. We verified that the absence of the Dectin-1 receptor resulted in a lower suppressive capacity of MDSCs, evidenced by a lower percentage of suppression on CD4^+^ and CD8^+^ T lymphocytes (**Figures 10A, B**). TLR2 absence in MDSCs had little effect on T-cell suppression, which could be detected only in the T CD4^+^ lymphocyte population, which presented an elevated proliferation index when cultured with MDSCs TLR2KO in comparison to the cultures with WT MDSCs (**Figures 10C, D**). This same pattern was observed in experiments addressing TLR4-deficient MDSCs, where only TCD4^+^ lymphocytes were slightly more proliferative when cocultured with MDSCs TLR4KO than when exposed to WT MDSCs (**Figures 10E, F**).

## 4 Discussion

In the present work, we demonstrated the relative role of three different PRRs expressed by MDSCs in the immunosuppression that characterizes the severe forms of pulmonary PCM. Using *in vitro* and *in vivo* experiments we showed that Dectin-1, TLR2, and TLR4 act to favor the suppressive activity of MDSCs, which is diminished after PRR blockade by monoclonal antibodies or when experiments are carried out with cells derived from knockout animals for these receptors. The *in vivo* experiments should be considered the most important results, as they best represent the complexity of infection and the possible outcomes of the absence of PRRs in animals (including the absence on the surface of MDSCs). In this context, we showed that Dectin-1, TLR2, and TLR4 are important for the basic activity of MDSCs in a fungal disease, bringing to light discoveries recently evaluated in the context of cancer and other diseases.

In the tumor environment, damaged cancer cells produce redundant damage-associated molecular patterns (DAMP) recognized by specific PRRs on MDSCs (Man and Jenkins, 2022). Interestingly, a link between specific PRRs (mainly TLRs) and cancer-associated microorganisms has been described, including *Helicobacter pylori* and Epstein–Barr virus in gastric cancer (Shehab et al., 2019; Dooyema et al., 2022), hepatitis B virus and hepatitis C virus in hepatocellular carcinoma, human papillomavirus in cervical cancer (Hasan et al., 2013), and also gut microorganisms involved in dysbiosis such as *Bacteroides fragilis* (Round et al., 2011) in pancreatic cancer and colorectal carcinoma (Fan et al., 2021). The data above emphasizes that the presence and concentration of DAMPs and PAMPs can contribute differently to the effects on the host immune system and possibly to therapy outcomes.

PRRs represent the primary MDSC receptors for integrating signals from pathogens or damaged cells. By recognizing different ligands, PRRs orchestrate MDSC immunosuppressive function, survival, migration, accumulation, differentiation, and soluble molecule release, thus exerting protumor or antitumor effects in mice and humans (Wang et al., 2023). In cancer, the corresponding signal transduction after PRR stimulation amplifies the effect of the immunosuppressive response of MDSCs by producing a variety of proteins, including arginase-1, iNOS, IDO-1, prostaglandin E2, PD-L1, CD40, TNF-α, IL-1β, IL-6, cyclooxygenase-2, and others. Together, these molecules create an immunosuppressive tumor microenvironment with reduced antigen presentation and reduced T-cell or natural killer cell activation, proliferation, and cytotoxicity (Jang et al., 2020). Therefore, understanding the effects of signaling via diverse PRRs on MDSCs recruited to the inflammatory site in infectious diseases may reveal the relative contribution of each receptor in the immunoregulation that controls disease severity.

An important function of C-type lectin receptors for MDSCs activity has been revealed in a study using human samples and animal models of *A. fumigatus* and *C. albicans* infections (Rieber et al., 2015). Likewise, our group has previously revealed that in murine PCM IDO-1 produced by MDSCs needs the expression of Dectin-1 for adequate activation and suppressive activity (Kaminski et al., 2023; Preite et al., 2023). In cancer, fungal-derived β-glucan (a major ligand of Dectin-1) has been shown to induce the differentiation of monocytic MDSCs into a more mature population expressing a CD11c^+^F4/80^+^ phenotype and impaired iNOS and ARG-1 production via the Dectin-1 pathway *in vitro*, thereby reducing tumor progression in a mouse lung cancer model (Tian et al., 2013). In agreement, we showed that Dectin-1 absence influences nitrotyrosine production by MDSCs after *P. brasiliensis* infection. In this context, our results with nitrotyrosine and previous observations regarding the role of nitrogenous compounds in immune responses (Pinke et al., 2016; Luo et al., 2023), cells of very close origins, such as neutrophils and polymorphonuclear MDSCs, can use the same mechanisms, with the result for the host being either beneficial or harmful, depending on the cell type. For example, NO-producing neutrophils have been shown to confer protection against *Chlamydia psittaci* in mouse lung infection (Luo et al., 2023) while MDSCs-producing nitrotyrosine contributes to severe paracoccidioidomycosis (Preite et al., 2023). Moreover, it was previously shown that mast cells that phagocytose *C. albicans* produce NO through mechanisms involving Dectin-1 (Pinke et al., 2016), which is in agreement with our results with MDSCs.

We have previously shown the important effects of Dectin-1 absence in host immunity against *P. brasiliensis* infection. It was shown that the absence of Dectin-1 impaired the production of T-helper type 1 (Th1), Th2, and Th17 cytokines and the activation and migration of T-cells to the site of infection. Besides, compared with WT mice, the fungal infection of Dectin-1KO mice was more severe, resulting in enhanced tissue pathology and mortality rates (Loures et al., 2014). In another study, we have also shown that Dectin-1 deficiency causes a decrease in the total number of MDSCs recruited to the lungs only 72 hours after fungal infection, with no differences after 2 and 8 weeks (Kaminski et al., 2023).

The vast majority of studies regarding MDSCs and these innate receptors were conducted in the context of cancer and it has been shown that TLR1/2 agonists in MDSCs can act controversially. The TLR1/2 agonist Pam3CSK4 can promote the differentiation of monocytic MDSCs into M2-type macrophages with highly immunosuppressive functions in patients with colon, prostate, pancreas, or liver cancer (Wang et al., 2015); in melanoma, Pam3CSK4 upregulated the expression of PD-L1 (CD274) on immature myeloid cells (Fleming et al., 2019), in agreement to our findings presented here. Instead, in a model of hepatocellular carcinoma, Pam3CSK4 facilitates M-MDSCs polarization toward mature macrophages and dendritic cells that prevent tumor progression (Li et al., 2022). In MDSCs, TLR2 activation with bacterial lipoproteins favors cancer growth by enhancing MDSC recruitment, survival, and accumulation to the tumor site in mouse lung cancer, fibrosarcoma, lymphoma, melanoma, and colorectal cancer models and is also correlated with bad prognosis in human colorectal cancer tissues (Maruyama et al., 2015; Shime et al., 2017; Hangai et al., 2021). Considering that TLR2 absence led to a reduction in MDSCs expressing nitrotyrosine and that nitrotyrosine expression by MDSCs is associated with severe paracoccidioidomycosis in mice (Preite et al., 2023), it would be intuitive to assume that blocking TLR2 could contribute to reducing the deleterious effects mediated by nitrotyrosine^+^ MDSCs in PCM. However, another previous study by our group revealed that TLR2 deficiency results in increased Th17 immunity associated with a diminished expansion of Treg cells and increased lung pathology due to unrestrained inflammatory reactions (Loures et al., 2015). Therefore, TLR2 deficiency would only be therapeutic if directly targeted to MDSCs at an infectious site, otherwise, it could be harmful to the host.

The role of TLRs in MDSC activity has also been investigated in other disease processes (Arora et al., 2010; Ray et al., 2013; Dorhoi and Du Plessis, 2017; Oliveira et al., 2021). Interestingly, it has been shown that TLR2 agonists, like those present in *S. aureus*, can favor the differentiation of monocytes into MDSCs, leading to their accumulation in the skin lesions (Skabytska et al., 2014; Dorhoi and Du Plessis, 2017) of patients. Recently we have also shown that TLR2 and TLR4 signaling is important for IDO-1 production by MDSCs during murine PCM (Kaminski et al., 2023). Here, we showed that such signaling could also affect the expression of other immunosuppressive molecules produced by MDSCs in systemic fungal disease. Considering the effects of TLR2 on the host infected with *P. brasiliensis*, Loures et al. (2009) revealed that, *in vivo*, TLR2-deficient mice presented increased lung pathology due to unrestrained inflammatory reactions (Loures et al., 2009). Thus, the deficiency in the activity of MDSCs through the reduced production of immunosuppressive molecules in TLR2KO mice presented here could contribute to this unfavorable outcome for the host. Of note, TLR2 absence did not affect the recruitment of MDSCs towards the infected lungs in mice (Kaminski et al., 2023).

In tuberculosis, which enhances lung cancer risk (Ho et al., 2021), *Mycobacterium bovis* Bacille–Calmette–Guerín (BCG) infection upregulated PD-L1, CD40, and CD69 via induced expression of TLR2 and TLR4 in both M-MDSCs and PMN-MDSCs in mice, also upregulating iNOS expression, leading to an increased NO production necessary for the suppression of T-cells in BCG-infected mice (John et al., 2019). In the context of *C. albicans* infection, it has been demonstrated that the absence of TLR2 can increase the survival of neutrophils (Tessarolli et al., 2010). Given that PMN-MDSCs and neutrophils share a common origin, it is likely that the increase in PD-L1-expressing PMN-MDSCs observed in **Figure 4D** and **Supplementary Figure 5** is due to the absence of TLR2 which caused an increased survival of these cells in the infected lungs. Regarding TLR4 absence in the context of *P. brasiliensis* infection, *in vivo*, previous studies from our group revealed that TLR4-deficient mice developed reduced fungal burdens, but also decreased levels of pulmonary NO, proinflammatory cytokines, and antibodies, contributing to the inability of the host to clear totally their diminished fungal burdens (Loures et al., 2010). Alike the findings concerning TLR2, a comparison between WT and TLR4KO animals revealed no differences in the recruitment of leukocytes to the lungs after 72 h, 2 weeks, and 8 weeks of *P. brasiliensis* infection (Kaminski et al., 2023).

Concerning T-cell suppression the results observed here in coculture experiments confirm the strongest effect of Dectin-1 absence on the activity of MDSCs, in which the defects in the production of anti-inflammatory mediators were largely reflected in the reduced ability to suppress the proliferation of CD4^+^ and CD8^+^ T lymphocytes. Likewise, a small effect of TLR2 and TLR4 absence in MDSCs on the production of immunosuppressive molecules was reflected in a small effect on the suppression of CD4^+^ T lymphocytes, with no effect on CD8^+^ T populations. Although our primary focus was to investigate the role of MDSC receptors Dectin-1, TLR2, and TLR4 in murine PCM it is important to note that the suppressive action of MDSCs was independent of the presence of the fungus in some *in vitro* experiments. Notably, *in vivo*, the fungal infection and its associated cytokine milieu are the primary stimuli for the generation of MDSCs in mice (Preite et al., 2023). This discrepancy could explain why the effects observed *in vitro* do not exactly replicate *in vivo*. The difference between testing the absence of a molecule in an isolated environment and testing it in a complex environment further complicates comparisons. *In vitro*, cells are tested in the well of a culture plate. These cells are generated in a controlled setting. In contrast, *in vivo* experiments involve cells generated in an infected mouse. The so-established environment is rich with various stimuli from different cells. Additionally, *in vivo*, MDSCs interact with yeast after myelopoiesis is triggered by the host’s immune system stimuli. *In vitro*, MDSCs are generated by specific stimuli, such as IL-6 and GM-CSF, in an adapted culture medium.

The present work, together with findings from previous studies of our group, indicates that Dectin-1, TLR2, and TLR4 are important PRRs for the activity of MDSCs in pulmonary PCM, as already demonstrated in cancer and other diseases, such as other fungal, bacterial, and viral infections (Arora et al., 2010; Tessarolli et al., 2010; Ray et al., 2013; Dorhoi and Du Plessis, 2017; Wang et al., 2023). Given the few data available to date, our studies help to clarify the complex network of molecules used in the development and suppressive activity of MDSCs in PCM. We believe that this study is an important additional knowledge regarding the suppressive mechanisms that control PCM and helps to explain the therapeutic difficulties to overcome the immunosuppression associated with the severe forms of the disease.

## Data availability statement

The original contributions presented in the study are included in the article/**Supplementary Material**. Further inquiries can be directed to the corresponding author.

## Ethics statement

The animal study was approved by Comissão de Ética no Uso de Animais (CEUA/UNIFESP) - Ethics Committee on Animal Experiments of the Federal University of São Paulo-UNIFESP (Protocol Number 2135170220). The study was conducted in accordance with the local legislation and institutional requirements.

## Author contributions

VK: Conceptualization, Data curation, Investigation, Methodology, Validation, Visualization, Writing – original draft, Writing – review & editing. BB: Visualization, Writing – original draft, Writing – review & editing, Methodology. BS: Visualization, Writing – original draft, Writing – review & editing. NP: Visualization, Writing – original draft, Writing – review & editing. VC: Visualization, Writing – original draft, Writing – review & editing, Resources. FL: Resources, Visualization, Writing – original draft, Writing – review & editing, Conceptualization, Funding acquisition, Project administration, Supervision.
