# Supplementary material for: MDSCs use a complex molecular network to suppress T-cell immunity in a pulmonary model of fungal infection
Source: Front Cell Infect Microbiol. 2024 Jul 5;14:1392744. doi: 10.3389/fcimb.2024.1392744 (PMC11257977; doi:10.3389/fcimb.2024.1392744)
Supplement: Supplementary file 1 [file DataSheet_1.pdf]

Supplementary Information

MDSCs use a complex molecular network to suppress T-cell immunity in a pulmonary model of fungal infection

Valéria de Lima Kaminski[1]; Bruno Montanari Borges[1]; Bianca Vieira dos Santos[1]; Nycolas Willian Preite[1]; Vera Lucia Garcia Calich[2]; Flávio Vieira Loures[1]

[1] Institute of Science and Technology, Federal University of São Paulo – UNIFESP, São José dos Campos, São Paulo, Brazil.

[2] Department of Immunology, Institute of Biomedical Sciences, University of São Paulo – USP, São Paulo, Brazil.

* Corresponding Author: Flávio Vieira Loures

E-mail: loures@unifesp.br

Authors' e-mail addresses:

Valéria de Lima Kaminski (VLK): valeria.lkaminski@gmail.com

Bianca Vieira dos Santos (BVS): bsantos25@unifesp.br

Nycolas Willian Preite (NWP): preite@unifesp.br

Bruno Montanari Borges (BMB): bmborges@unifesp.br

Vera Lúcia Garcia Calich (VLGC): vlcalich@icb.usp.br

Supplementary Figure 1

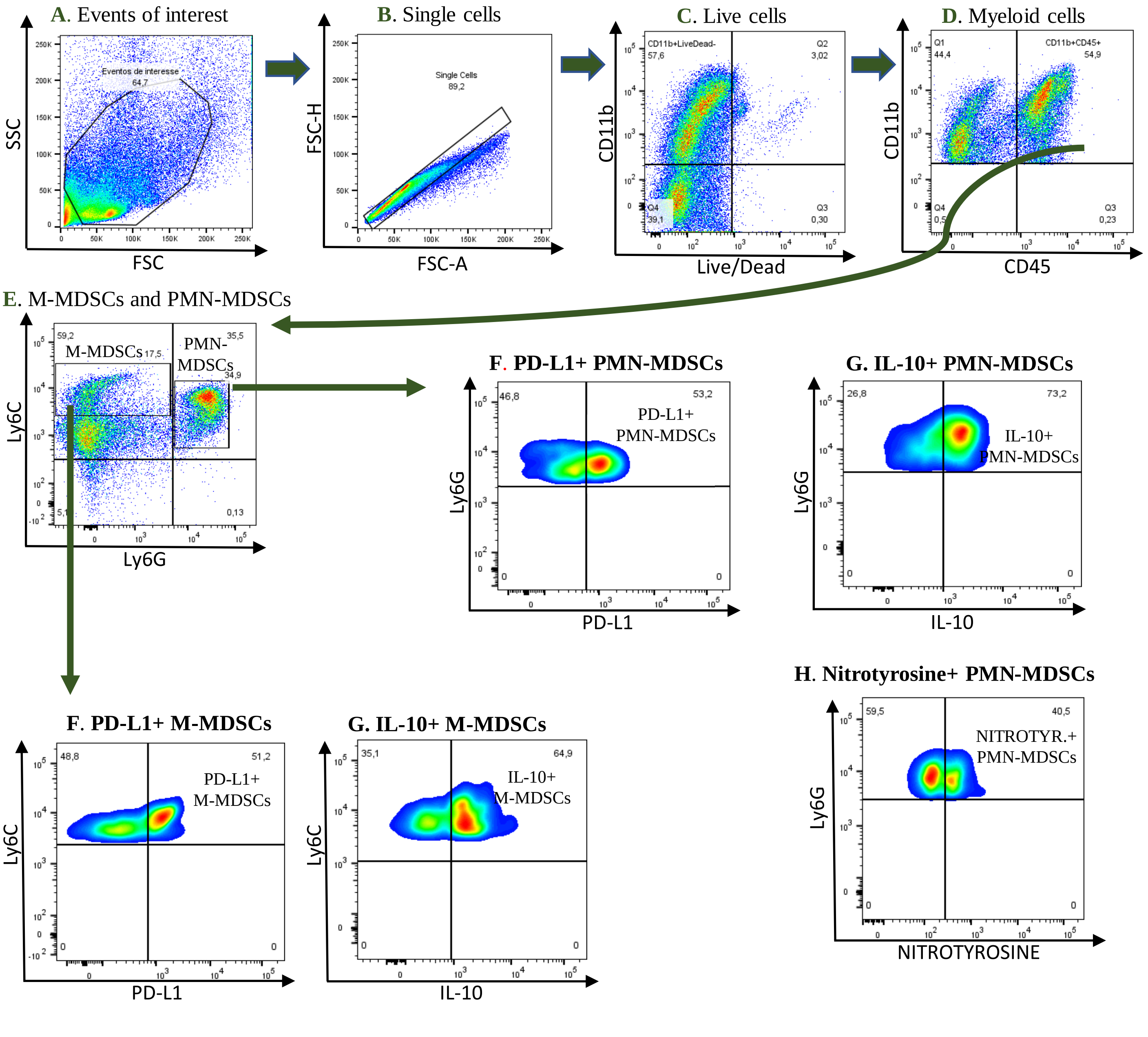

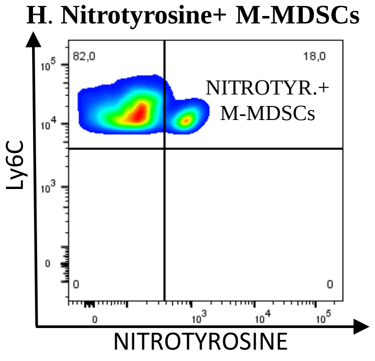

Figure Legend of Suppl. Fig 1. Immunophenotyping of MDSCs. This figure shows these cells' gating strategy for identifying MDSCs and PD-L1, IL-10, and NITROTYROSINE expression. **A –** Cells were gated by FSC/SSC analysis; **B** – Doublets were excluded; **C** – Live cells were selected; **D** – Myeloid cells were selected; **E**– Populations of M-MDSCs (Live/Dead-$CD45^{+}CD11b^{+}LY6G^{-}LY6C^{hi}$) and PMN-MDSCs ($CD45^{+}CD11b^{+}LY6G^{+}LY6C^{low}$) generated *in vitro* or recovered from infected lungs were assessed. **F** – Selection of M- or PMN-MDSCs **PD-L1+. G –** Selection of M- or PMN-MDSCs **IL-10+**. **H** - Selection of M- or PMN-MDSCs **nitrotyrosine+**. Samples were run on a FACS Lyric flow cytometer, and 100,000 events were acquired from in vivo experiments, and 50,000 events were acquired for in vitro experiments. Analysis was performed using FlowJo software (Tree Star).

Supplementary Figure 2

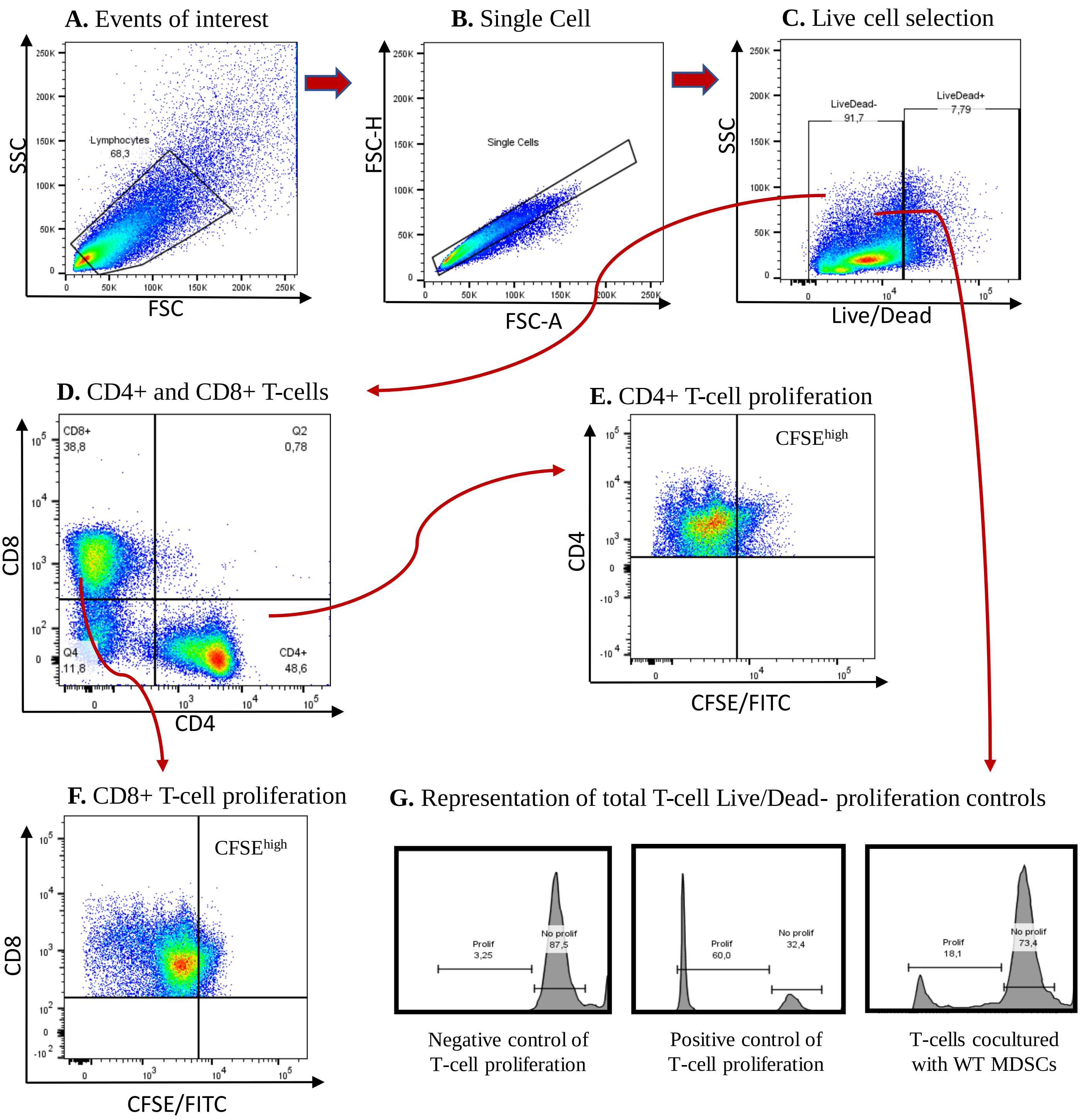

Figure Legend of Suppl. Fig 2. Lymphoproliferation. Magnetically sorted T-cells from naïve mice spleens were labeled with CFSE, activated with anti-CD3/CD28 and cultured for 4 days in the presence or absence of MDSCs (exposed or not to *P. brasiliensis* yeasts). One million T cells were placed by well in a 96-well U-bottom plate. **A – F**: T-cells were then characterized as CD4+ or CD8+, and CFSE fluorescence was assessed through the FITC channel. **G**: representation of T-cell proliferation controls in total Live/Dead- cells. Samples were run on a FACS Lyric flow cytometer, and 50,000 events were acquired in samples. Analysis was performed using FlowJo software (Tree Star). Numbers 6 and 7 represent the controls of T-cell proliferation.

Supplementary Figure 3.

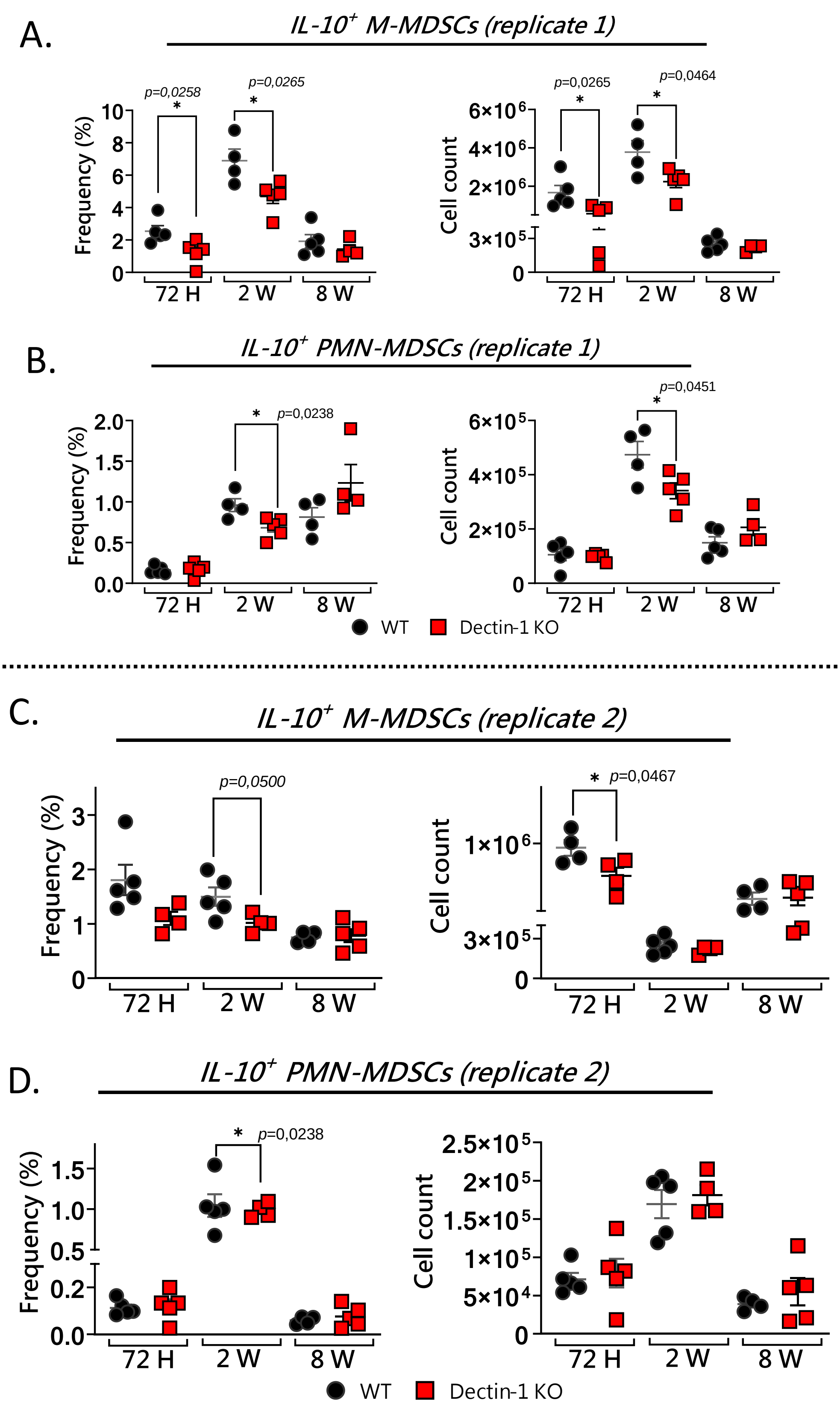

Figure Legend of Suppl. Fig 3. (**A** and **B**) WT and Dectin-1KO mice were intratracheally infected with 1×10^6 *P. brasiliensis* yeasts. Lungs were collected after 72 h, two weeks, and eight weeks of infection. Adequate antibodies conjugated to fluorochromes were used to characterize MDSC subpopulations, as shown in Suppl. Fig. 1. The frequency and total cell count of lung-infiltrating M-MDSCs (**A** and **C**) and PMN-MDSCs (**B** and **D**) positive for **IL-10** 72 h, two weeks, and eight weeks post-infection were determined by comparing WT with Dectin-1 KO. The data represent three independent experiments with 3–5 mice each. For comparisons between the two groups, the mean ± SEM was obtained and analyzed by the unpaired Student's t-test. Differences were considered significant at $*p < 0.05$. *These data are replicates of the experiment shown in chief Fig 2.*

Supplementary Figure 4. The experiments presented below are the replicates of the *in vivo* experiment shown in **Figure 3**, using 3-5 animals.

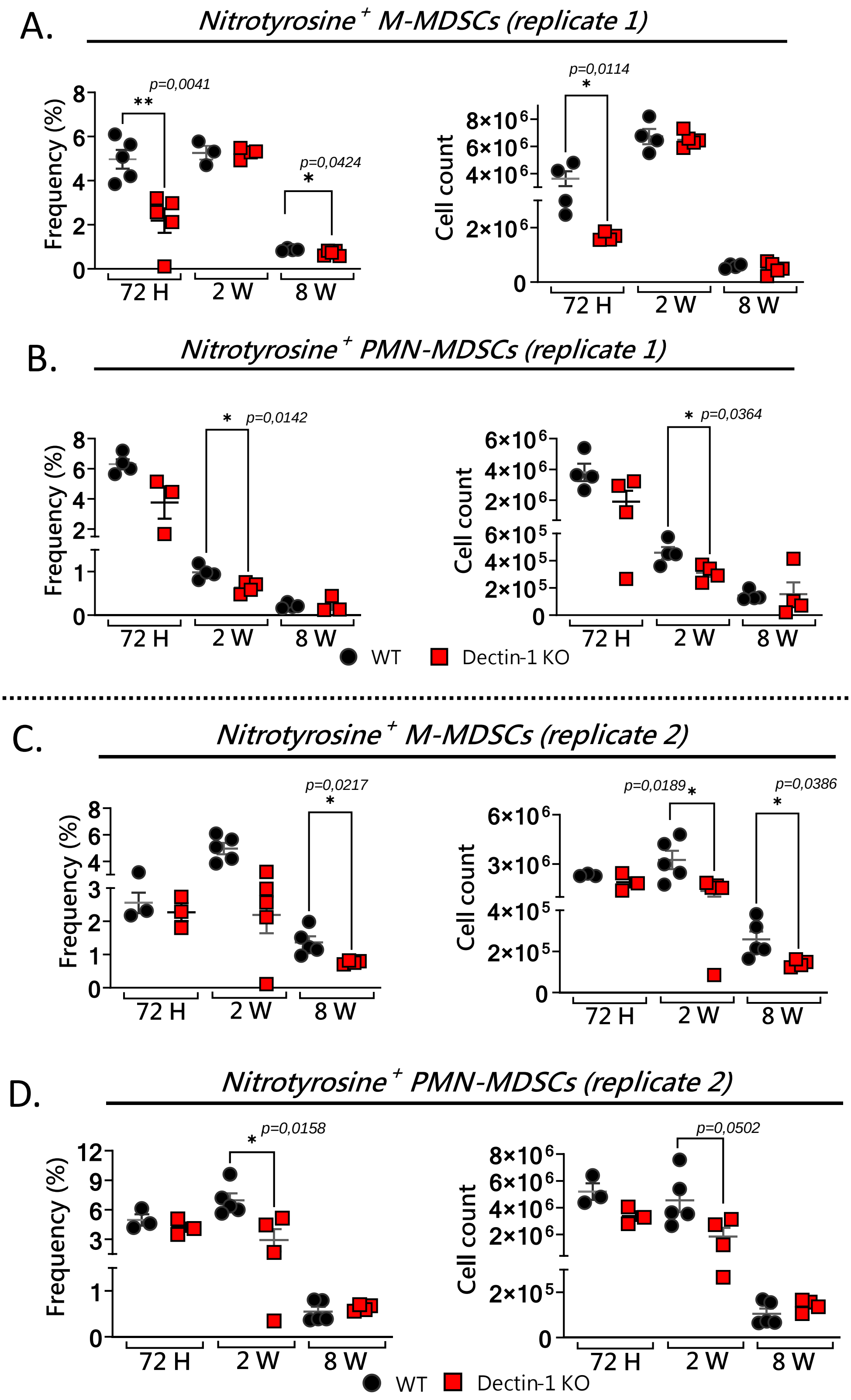

Figure Legend of Suppl. Fig 5. (**A** and **B**) WT and Dectin-1KO mice were intratracheally infected with 1×10^6 *P. brasiliensis* yeasts. Lungs were collected after 72 h, two weeks, and eight weeks of infection. Adequate antibodies conjugated to fluorochromes were used to characterize MDSC subpopulations, as shown in Suppl. Fig. 1. The frequency and total cell count of lung-infiltrating M-MDSCs (**A** and **C**) and PMN-MDSCs (**B** and **D**) positive for **nitrotyrosine** 72 h, two weeks, and eight weeks post-infection were determined by comparing WT with Dectin-1 KO. The data represent three independent experiments with 3–5 mice each. For comparisons between the two groups, the mean ± SEM was obtained and analyzed by the unpaired Student's t-test. Differences were considered significant at $*p < 0.05$. *These data are replicates of the experiment shown in chief Fig 4.*

Supplementary Figure 5.

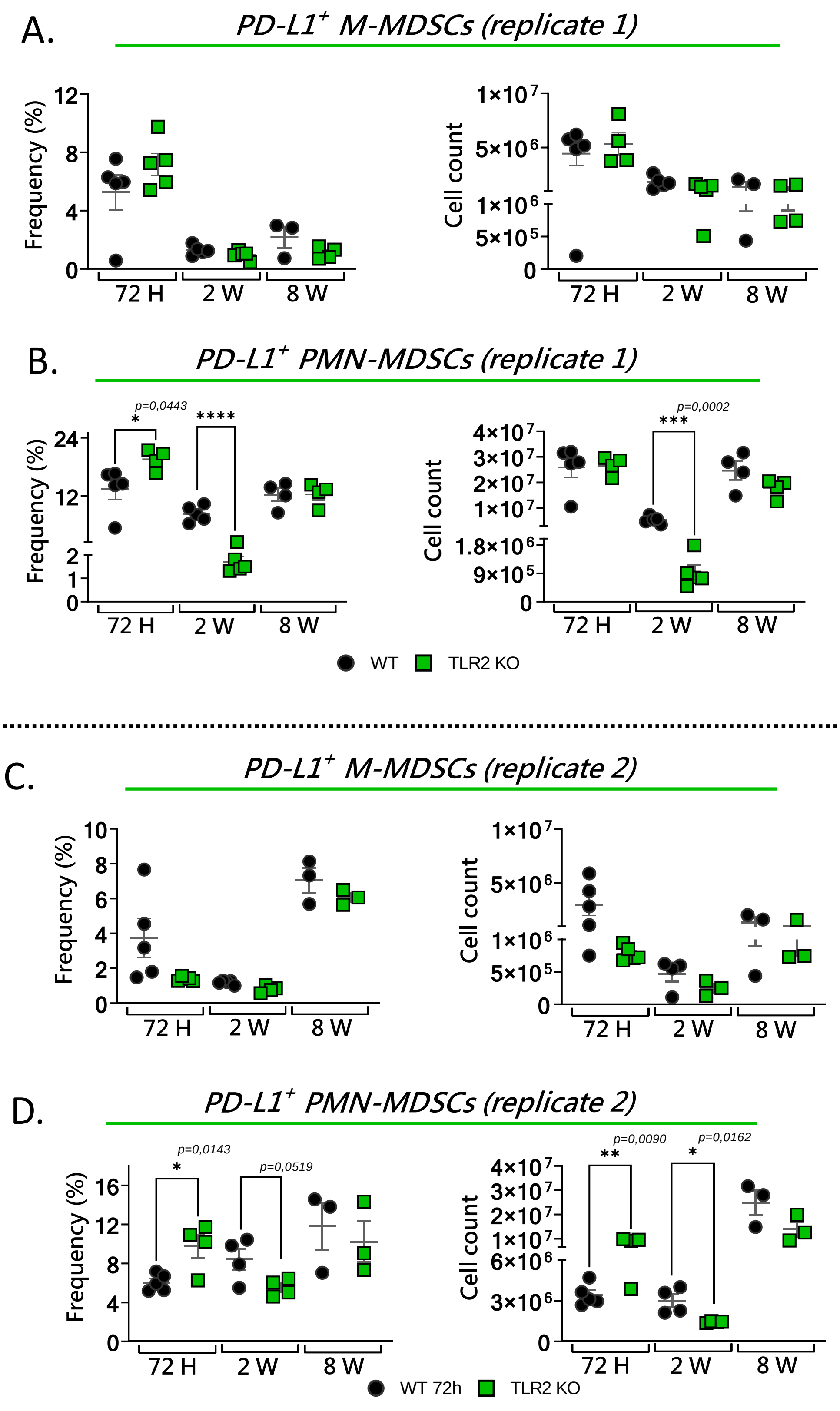

Figure Legend of Suppl. Fig 5. (**A** and **B**) WT and TLR2KO mice were intratracheally infected with 1×10^6 *P. brasiliensis* yeasts. Lungs were collected after 72 h, two weeks, and eight weeks of infection. Adequate antibodies conjugated to fluorochromes were used to characterize MDSC subpopulations, as shown in Suppl. Fig. 1. The frequency and total cell count of lung-infiltrating M-MDSCs (**A** and **C**) and PMN-MDSCs (**B** and **D**) positive for **PD-L1** 72 h, two weeks, and eight weeks post-infection were determined by comparing WT with Dectin-1 KO. The data represent three independent experiments with 3–5 mice each. For comparisons between the two groups, the mean ± SEM was obtained and analyzed by the unpaired Student's t-test. Differences were considered significant at $*p < 0.05$. *These data are replicates of the experiment shown in chief Fig 4.*
